# Zwitterionic Poly(Carboxybetaine Methacrylate)s in Drug Delivery, Antifouling Coatings, and Regenerative Tissue Platforms

**DOI:** 10.3390/ma18194514

**Published:** 2025-09-28

**Authors:** Theodore Sentoukas, Wojciech Walach, Katarzyna Filipek, Barbara Trzebicka

**Affiliations:** Centre of Polymer and Carbon Materials, Polish Academy of Sciences, M. Curie-Skłodowskiej 34, 41-819 Zabrze, Poland; tsentoukas@cmpw-pan.pl (T.S.); wwalach@cmpw-pan.pl (W.W.); kfilipek@cmpw-pan.pl (K.F.)

**Keywords:** PCBMA, antifouling, superhydrophilic polymer, zwitterionic polymer

## Abstract

Poly(carboxybetaine methacrylate)s (PCBMA) belongs to a class of zwitterionic polymers that offer promising alternatives to polyethylene glycol (PEG) in biomedical applications. This review highlights how the unique zwitterionic structure of PCBMA dictates its strong antifouling behavior, low immunogenicity, and sensitivity to environmental stimuli such as pH and ionic strength. These features make PCBMA promising for designing advanced systems suited for complex biological environments. This review describes PCBMA-based materials—ranging from hydrogels, nanogels, and surface coatings to drug carriers and protein conjugates—and critically evaluates their performance in drug delivery, tissue engineering, diagnostics, and implantable devices. Comparative studies demonstrated that PCBMA consistently outperformed other zwitterionic polymers and PEG in resisting protein adsorption, maintaining bioactivity of conjugated molecules, and ensuring long circulation times in vivo. Molecular dynamics simulations provide additional information into the hydration shells and conformational behaviors of PCBMA in aqueous dispersions. These insights underscore PCBMA’s broad potential as a promising high-performance material for next generation healthcare technologies.

## 1. Introduction

Polymers for biomedical applications remain a constant challenge. Research has focused on modifying polymer surfaces to reduce protein binding and immunogenicity, developing biodegradable materials that degrade safely into non-toxic compounds, and designing targeted delivery systems that evade the immune system and localize to diseased tissues.

Polyethylene glycol (PEG) is the best-known and most widely used polymer for such purposes. Its biomedical applications advanced significantly in the 1970s with the introduction of PEGylation, where PEG chains were attached to liposomes or biological macromolecules. PEGylated drugs achieved longer circulation times, reduced immunogenicity and improved efficacy, revolutionizing drug delivery. PEG-based hydrogels created biocompatible scaffolds for cell growth, while PEG coatings improved medical devices by reducing thrombosis and infection risks [1,2,3,4,5,6,7].

Despite its status as the “golden standard,” PEG has some limitations. Its stealth properties on nanoparticles often reduce cellular uptake, impairing therapeutic efficacy. PEG can also elicit anti-PEG antibodies, leading to allergic reactions [8,9,10,11,12,13]. In localized delivery systems such as hydrogels and microrobotic carriers, PEG contributed structural stability and biocompatibility but restricted drug loading, responsiveness to biochemical stimuli, and penetration into tissues [14,15].

These drawbacks started the investigation of alternative hydrophilic polymers, including poly(2-oxazoline)s, poly(hydroxypropyl methacrylamide) (PHPMA), and polybetaines. Among them, polybetaines such as polysulfobetaines, polycarboxybetaines, and polyphosphobetaines stand out as zwitterionic polymers combining positive and negative charges within the same repeat unit. Their hydrophilicity, antifouling capacity, biocompatibility, and thermal and chemical stability make them strong PEG substitutes [16,17,18]. They resist nonspecific adsorption of proteins and bacteria, maintain stability under diverse conditions, and can respond to changes in pH and ionic strength. Zwitterionic polymers have been applied in biomedical devices, drug delivery, water treatment membranes, sensors, and tissue engineering. Challenges remain in achieving large-scale synthesis with controlled architectures and functionalities and current research aims to improve synthetic strategies and deepen understanding of their interactions with biological systems [19,20,21,22,23].

**Figure 1:**
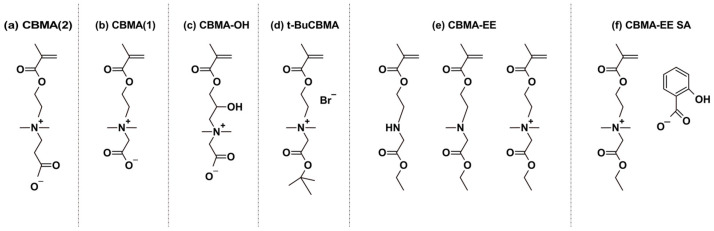
Different CBMA monomers (**a**) N,N-dimethyl((methacryloyloxy)ethyl) ammonium propiolactone, (**b**) 2-((2-(methacryloyloxy)ethyl)dimethylammonio)-acetate, (**c**) 2-((2-hydroxy-3-(methacryloyloxy)propyl)dimethylammonio)acetate, (**d**) 2-tert-butoxy-N-(2-(methacryloyloxy)ethyl)-N,N-dimethyl-2-oxoethanaminium, (**e**) carboxybetaine methacrylate ethyl ester (2°, 3°, and 4° CBMA-EE, respectively), and (**f**) N,N-dimethyl-N-(ethylcarbonylmethyl)-N-[2-(methacryloyloxy)ethyl] ammonium salicylate.

Within this group, poly(carboxybetaine methacrylate) (PCBMA) consists of methacrylate esters bearing quaternary ammonium and carboxylate groups, separated by either an ethylene or methylene spacer (Figure 1). This structure provides exceptional hydrophilicity, antifouling properties, and biocompatibility, making PCBMA more suitable for biomedical use than other zwitterionic polymers such as poly(2-(methacryloyloxy)ethyl phosphorylcholine) (PMPC) and poly(sulfobetaine methacrylate) (PSBMA).

This review summarizes PCBMA research, including monomers, synthesis methods, and the preparation of various architectures such as homopolymers, block copolymers, star-shaped polymers, nanogels, hydrogels, membranes, and surface-grafted coatings. We emphasize how the zwitterionic nature of PCBMA governs antifouling and environmental responsiveness, with applications in drug delivery, tissue engineering, and antifouling coatings. This review also discusses molecular dynamics studies of PCBMA macromolecules in aqueous solutions and highlights key issues that could guide the expansion of PCBMA across scientific and industrial fields.

## 2. Carboxybetaine Methacrylate Monomer: Synthesis and Properties

Several variants of carboxybetaine methacrylate have been described in the literature (Figure 1 and Table 1) due to the possibility of different linkages between the carboxyl group and the positively charged nitrogen atom in the carboxybetaine group. One of the earliest was N,N-dimethyl((methacryloyloxy)ethyl)ammonium propiolactone, reported by Liaw et al. [24]. This variation contains an ethylene group (C2 spacer) between the quaternary ammonium and the carboxyl pendant group and is referred to as CBMA(2) in this study (Figure 1a). Another way of synthesizing CBMA(2) monomer was later described by Lin et al. [25] (US patent 20140275614).

Carboxybetaine monomer with a tert-butyl protected carboxylic group (Figure 1d), (2-tert-butoxy-N-(2-(methacryloyloxy)ethyl)-N,N-dimethyl-2-oxoethanaminium), was reported by Cao et al. [26]. This compound was compatible with hydrophobic monomers in organic solvents and is referred to as t-BuCBMA in this study. Hydrolysis of the protecting ester group in t-BuCBMA leads to GLBT [27]. This CBMA(1) monomer contains a methylene group (C1 spacer) separating the quaternary ammonium and the carboxyl pendant group (Figure 1b), in contrast to the ethylene group in CBMA(2) [24]. It is referred to as CBMA(1) in this study.

An alternative variation of CBMA was reported by Cao et al. [28]. The obtained monomer [2-((2-hydroxy-3-(methacryloyloxy)propyl)dimethylammonio)acetate], shown in Figure 1c, is referred to as CBMA-OH in this study. CBMA-OH can reversibly alternate between an open carboxylate form (CBMA-OH) and a six-membered lactone ring (CBMA-Ring) through pH changes. CBMA-OH converts to the CBMA-Ring within two hours in acidic conditions, while the reverse reaction occurs rapidly under neutral or basic conditions. It showed minimal protein adsorption from both single protein solutions and undiluted human plasma, demonstrating ultralow-fouling characteristics.

Cheng et al. [29] reported an ethyl ester protected CBMA(1), N,N-dimethyl-N-(ethylcarbonylmethyl)-N-[2-(methacryloyloxy)ethyl]ammonium bromide, denoted as CBMA-EE (Figure 1e). A further modification by Cheng et al. [30] involved exchanging the bromide counter-ion with salicylate, yielding N,N-dimethyl-N-(ethylcarbonylmethyl)-N-[2-(methacryloyloxy)ethyl]ammonium salicylate, referred to as CBMA-EE SA in this study (Figure 1f).

## 3. Carboxybetaine Methacrylate Polymers and Their Properties

### 3.1. Homopolymers

PCBMA has been predominantly studied in the context of copolymers, gels, surface coatings, and conjugates with biological molecules. While much attention has been given to its properties and applications, studies focusing on the homopolymerization of PCBMA remain limited. The available reports mainly describe homopolymerization via reversible addition–fragmentation chain transfer (RAFT) or atom transfer radical polymerization (ATRP) methods, often followed by copolymerization with a second block or conjugation with biological molecules. This indicates a gap in research, specifically kinetics of polymerization of pure CBMA.

A summary of CBMA homopolymerization data is given in Table 2, providing key details such as the synthesis methods, molar masses, yields, specific synthesis parameters, and subsequent applications of the homopolymers, whether they were further used for block copolymerization or conjugation with biological molecules. The polymerization conditions include various initiators, catalysts, and solvent systems. Polymerization yields are typically high, often exceeding 80%. The synthesized PCBMA homopolymers exhibit a wide range of molar masses, from 2.5 kDa to nearly 100 kDa, depending on the polymerization technique and conditions. Techniques such as ATRP and RAFT generally provide good control over molar mass.

One of the earliest syntheses of CBMA homopolymers was performed by Liaw et al. [24] in 1997 using free radical polymerization of CBMA(2) in aqueous media (Table 2, entry 1). In water, the resulting PCBMA(2) formed dispersions of an ionically crosslinked transparent network. The addition of salts disrupted the ionic interactions within the polymer network, enhancing the solubility of the chains. The polymer showed significant water absorption due to the presence of carboxylate groups.

Most studies of PCBMA preparation focus on the resulting applications rather than providing in-depth analysis of the polymerization parameters. The exception is the study by Ning et al. [31] who, according to the reported RAFT kinetics results of CBMA(1), obtained well-controlled molar masses from 5 to almost 20 kg/mol. Lim et al. [32] discussed the difficulties of CBMA(1) homopolymers (Table 2, entry 17) and PCBMA(1)-b-PSBMA copolymer synthesis that came from macro-chain transfer agent (CTA) deactivation or self-assembly phenomena that affected chain growth. They also mentioned that these (co)polymers showed variances in yield due to the challenges posed by the amphiphilic nature of the polymers during synthesis. In [21], Lim et al. discussed RAFT homopolymerization and copolymerization of CBMA(1), (Table 2, entry 18). They highlighted that although high CBMA(1) monomer conversion was achievable, minor imbalances and variations in dispersity occurred, especially at high target DPs or under viscous conditions that hindered mixing. These imperfections influenced the ability of the copolymers to form fully controlled self-assembled structures, particularly those requiring a precise block length ratio.

Ma et al. [33] synthesized CBMA(1) homopolymers via free radical polymerization in mixed aqueous media (Table 2, entry 2) and investigated their ability to bind non-freezing water. PMPC, poly(carboxybetaine acrylamide) (PCBAA), and PSBMA zwitterionic polymers were also synthesized and compared with PEG. PMPC and PCBAA bound the largest number of non-freezing water molecules; 10.7–10.8 water molecules per repeating unit. This finding was attributed to the presence of the amide group in their structures, which enhanced water binding compared to the ester group in PCBMA and PSBMA. PEG, by comparison, binds significantly less non-freezing water molecules since zwitterionic hydration relies on strong electrostatic interactions, while PEG binds water mainly via weak hydrogen bonds.

Higaki et al. [34] synthesized CBMA(2) homopolymers of varying molar masses by RAFT polymerization (Table 2, entry 12) and examined their cononsolvency behavior in water/ethanol mixtures. PCBMA(2) dissolved well in pure water (0% ethanol), diluted ethanol (≤60% ethanol), and pure ethanol (100% ethanol). Aggregation occurred in intermediate ethanol ratios (62 to 80% ethanol), leading to the formation of large aggregates at 70% ethanol. At higher ethanol content (82 to 96% ethanol), the polymer became insoluble, precipitating as a viscous fluid. This cononsolvency behavior reflects the competitive hydrogen bonding and solvation interactions between water and ethanol, which destabilize the hydration layer around the polymer chains in specific mixtures. This behavior was attributed to lower association constants for water and ethanol compared to PMPC. Molecular modeling confirmed smaller association constants and a broader cononsolvency range for PCBMA compared to PMPC due to differences in electrostatic potentials, dipole moments, and van der Waals interactions.

Despite these advances, the literature on PCBMA homopolymers remains limited compared to PEG and other zwitterionic polymers. Systematic kinetic studies are scarce, with only isolated reports on RAFT kinetics; moreover, practical challenges such as macro-CTA deactivation and chain aggregation complicate reproducibility at high degrees of polymerization. Most studies report synthesis on small scales under controlled conditions and little is known about scalability, long-term storage stability, or degradation under physiological stress. Addressing these gaps will be essential to advance CBMA homopolymers beyond proof-of-concept laboratory demonstrations.

**Table 2:** CBMA homopolymers, molecular and physicochemical properties, and further use.

Entry	Polymerization Technique	M_n_ (kDa), (M_w_/M_n_)	Yield	Polymerization Conditions	Used For	Ref.
1	Free radical	-	93%	CBMA(2) and ACVA initiator in water	Homopolymer for aqueous solution studies	Liaw et al. [24]
2		33		CBMA(1) and AIBN initiator in water/methanol (4:1)	Homopolymer for water-binding studies	Ma et al. [33]
3		11.4		CBMA(1), AIBN, and 2-mercaptoethanol as chain transfer agents	Homopolymer	Kitano et al. [35]
4	ATRP	13.64	-	t-BuCBMA, 2-aminoethyl 2-bromoisobutyrate ATRP initiator, copper bromide [Cu(I)Br]/1,1,4,7,10,10-Hexamethyltriethylenetetramine (HMTETA) catalyst system in DMF; and hydrolysis in TFA	Conjugation with poly(lactic-co-glycolic acid)	Cao et al. [26]
5		12.4		CBMA-EE with ethyl 2-bromoisobutanoate (EBIB) initiator, CuBr, and PMDETA in methanol	Block copolymerization with CBMA(2)	Zhang et al. [36]
6		3.5	-	CBMA(2), EBIB initiator, and HMTETA/CuBr in 1:1 methanol–DMF	Block copolymerization with 2,2-di(acryloyloxy-1-ethoxy)propane-co 4,4-trimethylene dipiperidine) P(ADA-co-TMDP)	Ma et al. [37]
7		2.5 (1.04), 6.5 (1.17)	-	t-BuCBMA, N-hydroxysuccinimide (NHS)-terminated initiator, and Cu(I)Br/HMTETA catalyst system in DMF; hydrolysis of tert-butyl groups in TFA	Conjugation with α-chymotrypsin	Keefe et al. [38]
8		5.41 (1.03)	-	tBuCBMA monomer, NHS ester of 2-bromopropanoic acid as the ATRP initiator, and the Cu(I)Br/HMTETA catalyst system in anhydrous DMF; hydrolysis of tert-butyl groups in TFA	Conjugation with liposomes	Cao et al. [39]
9		35.5 (1.51)	-	CBMA(2) monomer, EBIB as initiator, and (CuBr)/(CuBr_2_)/2,2′-bipyridine (bpy) as catalysts in H_2_O/DMF	Functionalization with curcumin	Zhao et al. [40]
10		88.7 (1.32)		CBMA(2) monomer, EBIB initiator, and the CuBr/CuBr_2_/bpy catalyst system in H_2_O/DMF	Conjugation with LK7 enzyme	Zhao et al. [41]
11		8.1 (1.4), 11.9 (1.6), 20.9 (1.7), 30.8 (1.8), 38.7 (1.9)	-	CBMA(2) monomer, lysozyme-conjugated ATRP initiator, Cu(II)Br, sodium ascorbate (NaAsc), and HMTETA in octanol/water	Conjugation with lysozyme	Baker et al. [42]
12	RAFT	4.5, 9.8, 18, 32.4		CBMA(2), CPADB CTA, and 2,2′-azobis [2-(2-imidazolin-2-yl)propane] dihydrochloride as initiator in water–ethanol (2:1)	Homopolymer for cosolvency studies with ethanol	Higaki et al. [34]
13		13.4	-	CBMA(1) in water, 2-Cyano-2-methylethyl dithiobenzoate (CMEDTB) CTA, and ACVA in DMF	Copolymerization with (4-ethoxy-4′-methacrylamide) azobenzene	Shrivastava et al. [43]
14		DP = 60 and 90	-	CBMA(1), ACVA, and 4-CPADB CTA in water/DMF (4/1)	Block copolymerization with ethylhexyl acrylate PEHA	Matsuoka et al. [44]
15		11.9 (1.13), 25.2 (1.2), 33.6 (1.15), 64.5 (1.16)	-	CBMA(1), CPADB CTA, and ACVA in a water/DMF (4/1)	Block copolymerization with n-butyl acrylate (n-BA)	Murugaboopathy et al. [45]
16		9.5 (1.17)	83%	CBMA(1), Morpholine-functionalized-4-Cyano-4-(2-phenylethanesulfanyl-thiocarbonyl)sulfanylpentanoic acid CTA, and ACVA in pH 3.5 water	Copolymerization with HPMA	Ning et al. [31]
17		18.7 (1.07), 39.5 (1.10), 28.4 (1.06)	87% 80%, 90%	CBMA(1), CPADB CTA, and 2,2′-Azobis [2-(2-imidazolin-2-yl)propane] dihydrochloride (VA-044) radical initiator in water	Copolymerization with SBMA	Lim et al. [32]
18		49, 99, 198 DP	-	CBMA(1) and PETTC CTA (VA-044) in water/2,2,2-trifluoroethanol (TFE) (8/2)		Lim et al. [21]
19		6.49, 11.65	75%	CBMA(2), 2-cyanopropan-2-yl benzodithioate CTA, and AIBN as initiator in methanol	Homopolymer for complexation with siRNA	Peng et al. [46]
20a	Photo-RAFT	31.6	-	CBMA(2), sodium pyruvate (SP) photoinitiator, CPADB CTA, and AIBN in water/DMSO (9:1)	Homopolymer	Jazani et al. [47]
20b		80.2, 92.9	-	CBMA(2), sodium pyruvate (SP) photoinitiator, CT-CPADB-conjugated CTA, and AIBN in water/DMSO (9:1)	Conjugation with CT	

### 3.2. Copolymers and Their Self-Assembly

In this section, synthesis and properties of CBMA-based block copolymers, including their solution behavior, formation of nanoparticles, and functional properties, are presented. The ability of PCBMA nanostructures to encapsulate therapeutic agents is shown. Data from synthesis and molar masses are presented in Table 2 and Table 3.

Cao et al. [26] synthesized PLGA-b-PCBMA(1) copolymers by linking a tert-butyl-protected CBMA block to PLGA, followed by deprotection to yield amphiphilic copolymers of ~13.6 kDa (Table 2, entry 4 and Table 3, entry 1). The resulting materials had a PLGA/PCBMA weight ratio of ~10/1 as determined by ^1^H-NMR. When water was added to PCBMA(1)-b-PLGA organic solvent solution, the copolymers formed aggregates with a hydrodynamic diameter of 150 nm with a narrow size distribution. The nanoparticles remained stable for 13 h in various biological media, including phosphate-buffered saline (PBS) and fetal bovine serum (FBS), due to the strong hydration layer provided by the zwitterionic PCBMA(1) shell. In comparison, unmodified PLGA nanoparticles were heavily aggregated under the same conditions. No measurable cytotoxicity of PCBMA(1)-PLGA against HepG2 cells was observed after 24 h. Docetaxel, encapsulated with a payload of 1% w/w within the PCBMA(1)-b-PLGA nanoparticles, was released over 96 h.

**Figure 2:**
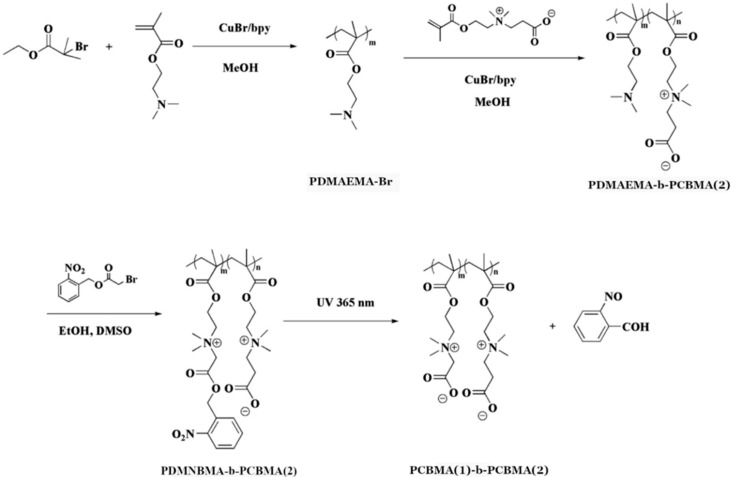
Synthesis of PDMNBMA-b-PCBMA(2) copolymers and their transformation after irradiation. Reproduced with permission [48]. Copyright 2014, American Chemical Society.

Jin et al. [48] introduced light-responsive functionalities to CBMA-based block copolymers. The authors prepared poly(N,N-dimethyl-N-(2-(methacryloyloxy)ethyl)-N-((2-nitrobenzyl)oxy)-2-oxoethanaminium bromide)-block-poly(carboxybetaine methacrylate) (PDMNBMA-b-PCBMA(2)) via sequential ATRP, as shown in Figure 2 and Table 3, entry 2. The PDMNBMA-b-PCBMA(2) copolymers were then complexated with negatively charged bovine serum albumin (BSA), forming polyion spherical structures of 162 nm diameter. The copolymer–protein complexes were pH-responsive due to the carboxylate groups of the PCBMA(2) block. The zeta potential was ~10 mV at physiological pH (7.4) and ~20 mV at pH 6.5. The complexes showed enhanced cellular uptake in acidic tumor environments. Irradiation of PDMNBMA-b-PCBMA(2)-BSA with UV-light caused cleavage of the o-nitrobenzyl groups of the PDMNBMA block and converted it into PCBMA(1), as shown in Figure 2. This resulted in the detachment of BSA associated with the PDMNBMA block, while maintaining the secondary structure of the protein.

Shrivastava et al. [43] developed novel amphiphilic block copolymers of PCBMA(1) and poly(4-ethoxy-4′-methacrylamidoazobenzene) (PEMAAB) with molar masses of 20.5, 22.0, and 30.0 kDa, containing azochromophore groups for further modification (Table 2, entry 13 and Table 3, entry 8). In aqueous solution at pH values of 2, 7, and 12, the copolymers formed vesicles. The formation and stability of vesicles depended on the ionic state of the copolymers. The size of the aggregates was in the range of 120–180 nm at all pH values and block ratios. When the copolymers were irradiated with UV light (360 nm), the reversible transformation of azochromophores from a nonpolar transform to a more polar cis form occurred. This isomerization affected the polarity of the copolymer, altering its hydrophilic–hydrophobic balance and transforming the copolymer vesicles into smaller micelles (30–40 nm).

Zhang et al. [36] prepared block copolymers of the PCBMA block with hydrophobic PCBMA-EE (Figure 1e) (Table 3, entry 4). PCBMA-EE-b-PCBMA/plasmid DNA complexes were prepared with a stable hydrophobic core of PCBMA-EE_50_, and a PCBMA corona that protects the structure from nuclease degradation. By varying the length of the PCBMA block, the balance between stability, DNA condensation, and cellular uptake was optimized. A long PCBMA block enhanced the stability of the polyplexes in DMEM with 10% FBS solution by creating a thick and uniform hydrophilic layer around the hydrophobic PCBMA-EE core. A short PCBMA block provided a thin shell, which can enhance cellular internalization, as the hydrophobic PCBMA-EE core is exposed, allowing interaction with the cell membrane.

PCBMAEE_50_-PCBMA_14_ provided a balance between shielding, efficient condensation, and uptake with low cytotoxicity. Upon hydrolysis, PCBMA-EE converted to zwitterionic PCBMA, which promoted pDNA release and further reduced cytotoxicity. Compared to conventional vectors, PCBMAEE_50_-PCBMA_14_ achieved up to 27-fold higher transfection efficiency than PEI (25 kDa) in COS-7 cells and 16-fold higher transfection than Lipofectamine^®^ 2000 in HUVECs. Even at only 5% of the standard DNA dose, it maintained ~25% of full-dose expression, corresponding to ~230-fold greater effectiveness than PEI. Hydrolyzed copolymers maintained >97% cell viability even at 100 μg/mL, which is much higher than Lipofectamine^®^ 2000 (54% viability) and PEI.

Xiu et al. [49] obtained structures of DEXTRAN-*g*-[PDMAEMA-*b*-PCBMA(2)], DEXTRAN-*g*-[PDMAEMA-*co*-PCBMA(2)], and DEXTRAN-*g*-(PDMAEMA-*b*-PSBMA) (Table 3, entry 6). Introduction of CBMA units to copolymers of DEXTRAN-g-PDMAEMA enhanced the biophysical properties of the gene carriers, improving stability in serum-containing media, reducing cytotoxicity, and enhancing cellular uptake. In COS7 cells, pristine DEXTRAN-g-PDMAEMA vectors suffered a ~62% reduction in transfection efficiency when serum increased from 10% to 30% FBS, while DEXTRAN-g-(PDMAEMA-b-PCBMA(2)) only lost ~28% and DEXTRAN-g-(PDMAEMA-b-PSBMA) maintained stable performance with no measurable decrease. Flow cytometry confirmed higher uptake for zwitterionic carriers, with DEXTRAN-g-(PDMAEMA-b-PSBMA)/pDNA reaching ~70% internalization at 10% FBS compared to ~54% for DEXTRAN-g-PDMAEMA and ~34% at 30% FBS compared to ~18% for DEXTRAN-g-PDMAEMA, while DEXTRAN-g-(PDMAEMA-b-PCBMA(2))/pDNA achieved ~63% internalization at 10% FBS and ~29% at 30% FBS under the same conditions. Cytotoxicity assays also showed that all zwitterionic-functionalized carriers maintained higher cell viability than DEXTRAN-g-PDMAEMA at equivalent N/P ratios. DEXTRAN-g-(PDMAEMA-b-PCBMA) and DEXTRAN-g-(PDMAEMA-b-PSBMA) exhibited significantly superior gene transfection efficiency and serum tolerance compared to DEXTRAN-g-(PDMAEMA-co-PCBMA), underscoring the importance of polymer architecture.

**Figure 3:**
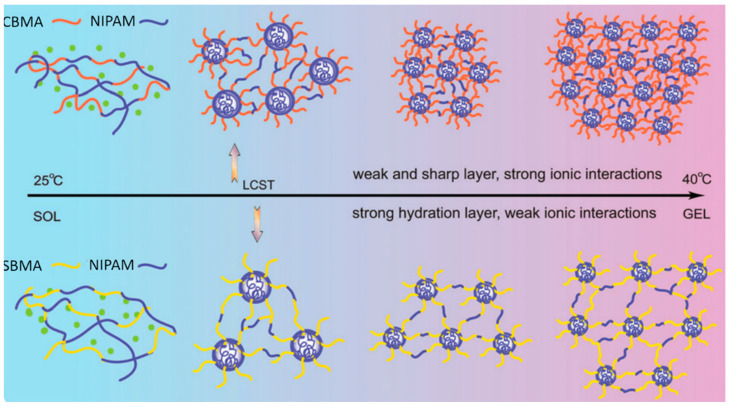
P(NIPAM-co-CBMA(2)) and P(NIPAM-co-SBMA) behavior in 25 °C to 40 °C. Reproduced with permission [50]. Copyright 2015, Royal Society of Chemistry.

Zhao et al. [50] synthesized thermoresponsive copolymers made of N-isopropylacrylamide (NΙPAM) and CBMA(2), using standard free radical polymerization in deionized water (Table 3, entry 18). P(NΙPAM-co-CBMA(2)) displayed a clear LCST of 34 °C, accompanied by a transition from a hydrophilic to a hydrophobic state and formation of a gel-like structure (Figure 3). P(NIPAM-co-SBMA), for comparison, exhibited a slightly elevated LCST (~34–36 °C) because SBMA side chains resisted NIPAM collapse, delaying aggregation (Figure 3). The copolymers showed weak phase transition and viscoelasticity, with G′ only ~1–8 Pa near 37 °C and limited recovery after significant strain. In contrast, CBMA units in P(NIPAM-co-CBMA) acted as ionic bridges, forming stable, elastic networks above the LCST (~34 °C). These networks displayed higher resilience, with G′ up to ~100–200 Pa and ~40% recovery after γ = 1000% oscillations at 37 °C. When cycled between 25 and 37 °C, CBMA gels fully restored elasticity through reversible hydration and ionic interactions, retaining nonfouling and thermoresponsive properties without loss of strength.

Matsuoka et al. [44] studied PCBMA(1)-b-PEHA copolymers at the air–water interface, synthesized via RAFT (Table 2, entry 14 and Table 3, entry 9). The PCBMA(1)-b-PEHA formed a dense brush monolayer beneath the water surface with a critical brush density of 0.30 chains/nm^2^. The thickness of the brush layer increased with increasing brush density due to the stretching effect. Unlike typical anionic and cationic brushes, the PCBMA(1) block layer increased its thickness upon salt addition due to transition from zwitterionic (neutral inner salt) to more ionic character.

Murugaboopathy et al. [45] obtained amphiphilic diblock copolymers composed of PCBMA(1) and poly(n-butyl acrylate) (P(n-BA)) via RAFT polymerization (Table 2, entry 15 and Table 3, entry 10). The copolymers had different lengths of the P(n-BA) block (DP = 47, 88, 104, and 254). PCBMA(1)-b-P(n-BA) copolymers were examined in both water and NaCl solutions to investigate their surface activity and self-assembly behavior. In pure water, these block copolymers exhibited weak surface activity, gradually lowering surface tension as their concentration increased. However, the addition of salt triggered a transition from surface-active to non-surface-active behavior. The critical micelle concentration of PCBMA(1)-b-P(n-BA) copolymers in water decreased with increasing salt concentration, reducing their surface activity in saline conditions. The hydrodynamic radius of the nanoparticles expanded from 63 to 86 nm as the NaCl concentration increased from 0.0 to 1.0 M.

Ning et al. [31] synthesized a poly(carboxybetaine methacrylate)-b-poly(hydroxypropyl methacrylate) (PCBMA(1)-b-PHPMA) via RAFT-mediated polymerization-induced self-assembly (PISA) (Table 2, entry 16 and Table 3, entry 11) for incorporation to calcite crystals to investigate occlusion phenomena. PCBMA(1)-b-PHPMA formed narrowly distributed nanoparticles (34.5 nm) in 1.5 mM Ca^2+^, indicating good colloidal stability for calcium carbonate formation. Ca^2+^ interacts with anionic carboxylate groups on the nanoparticle surface, leading to charge neutralization and reduced zeta potential. This caused increased aggregation or altering interactions with calcite surfaces. Crystals grown with PCBMA(1)-b-PHPMA exhibited typical rhombohedral morphology without polymer occlusion throughout calcite crystals and no weight loss up to 625 °C.

Ma et al. [37] synthesized block copolymers of CBMA(2) and [2,2-di(acryloyloxy-1-ethoxy)propane-co-4,4-trimethylenedipiperidine] [PCBMA(2)-b-P(ADA-co-TMDP)] via ATRP and Michael addition (Table 2, entry 6 and Table 3, entry 3). The authors also synthesized PDMAEMA-b-P(ADA-co-TMDP) copolymers to compare them with zwitterionic PCBMA(2)-analogs in various aspects such as protein adsorption, stability under different pH conditions, and drug delivery efficiency, particularly in terms of anticancer drug delivery. Both copolymers formed nanoparticles in PBS with a hydrodynamic diameter of 69 nm and a critical aggregation concentration (CAC) of 0.046 mg/mL for zwitterionic and 0.053 mg/mL for non-zwitterionic copolymers. A lower CAC indicates higher stability of PCBMA(2)-b-P(ADA-co-TMDP) nanoparticles due to thermodynamic favorability of aggregation.

Protein adsorption assays showed that at physiological pH (7.4), less than 15.6% of bovine serum albumin was adsorbed onto PCBMA-based particles after 4 h, compared to 18.1% on PDMAEMA analogs. Both blank carriers exhibited negligible cytotoxicity, maintaining ~100% viability of HepG2 and 3T3 cells. Upon drug loading, PCBMA-based carriers achieved a drug-loading efficiency (DLE) of 35.0% and drug-loading content (DLC) of 14.0 wt%, compared to 21.3% and 8.5 wt% for PDMAEMA analogs. The release of DOX was strongly pH-dependent, with cumulative release reaching 72.7% at pH 5.0 over 96 h, versus only 22.3% at pH 7.4. Cytotoxicity assays showed that the IC_50_ values of DOX-loaded PCBMA micelles were 0.76 μg/mL (HepG2) and 0.52 μg/mL (3T3), which were higher than free DOX (0.27 and 0.15 μg/mL, respectively). In vivo, DOX-loaded PCBMA-based carriers achieved a tumor inhibition rate of 89.3%, which is greater than free DOX (69.9%) and PDMAEMA analogs (60.2%), with reduced systemic toxicity and minimal weight loss in treated mice.

**Figure 4:**
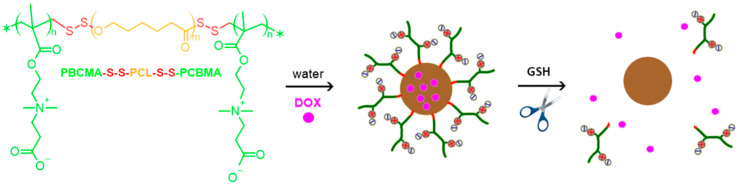
PCBMA(2)-b-PCL-b-PCBMA(2) copolymers, encapsulation of DOX, and intracellular behavior of the carrier. Reproduced with permission under the terms of the Creative Commons Attribution License (CC BY 4.0) [51]. Copyright 2019, MDPI.

Jiang et al. [51] synthesized poly(carboxybetaine methacrylate)-b-poly(ε-caprolactone)-b-poly(carboxybetaine methacrylate) (PCBMA(2)-b-PCL-b-PCBMA(2)) copolymers (Figure 4) (Table 3, entry 12). In PBS, PCBMA(2)-b-PCL-b-PCBMA(2) created spherical nanoparticles of an average hydrodynamic diameter of 87 nm with a narrow size distribution. The nanoparticles showed good stability and preserved their sizes in the presence of serum proteins, such as BSA and FBS, due to the antifouling properties of PCBMA(2). They also showed low cytotoxicity, as demonstrated in assays with HepG2 cells and high cell viability over a range of concentrations. The nanoparticles served as carriers of DOX (Figure 4) with 15% DLC and 41% DLE. The drug was released rapidly in a reducing cellular environment and exhibited high antitumor activity against HepG2 cells. Under physiological conditions (pH 7.4) in the presence of dithiothreitol (DTT), approximately 61% of the DOX was released after 48 h and only 31% without DTT.

Lim et al. [32] synthesized PCBMA(1)-b-PSBMA copolymers using RAFT (Table 2, entry 17 and Table 3, entry 13). In water, due to the upper critical solution temperature behavior of the PSBMA block, PCBMA(1)-b-PSBMA aggregated at room temperature (25 °C) to spherical nanoparticles of well-defined size distribution, while the increase in temperature to 60 °C led to particle disassembly. In their next work, Lim et al. [21] synthesized PCBMA(1)-b-PSBMA via one-pot RAFT copolymerization. The copolymer was compared with poly[2-(methacryloyloxy)ethyl phosphorylcholine)]-b-poly(sulfobetaine methacrylate) (PMPC-b-PSBMA) and PSBMA-b-PCBMA(1)-b-PSBMA copolymers. The authors synthesized di- and triblock copolymers with PCBMA blocks of DP in the range of 46 to 198 (Table 2, entry 18 and Table 3, entry 14). The behavior of all copolymers in aqueous solutions was mainly ruled by interactions between PSBMA blocks. Triblock copolymers with PSBMA end blocks at temperatures below 10 °C formed monodisperse particles with a flower-like structure.

**Figure 5:**
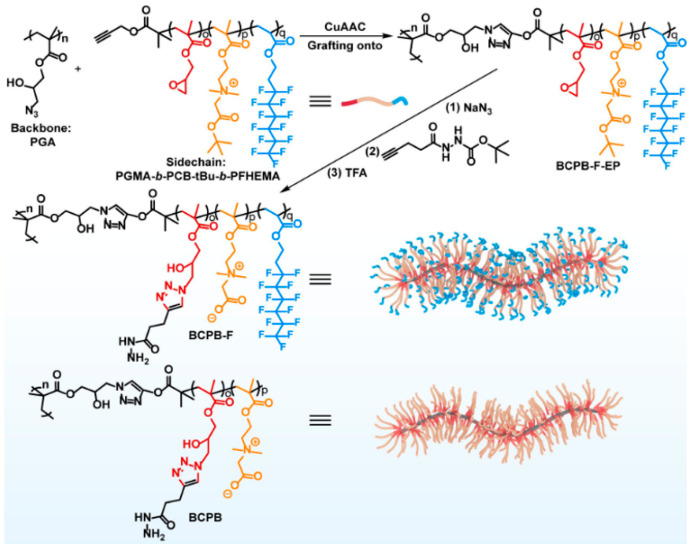
Synthetic route of PGA-g-(PGMA-b-PCBMA-b-PFHEMA) graft copolymers and their non-fluorinated analogs. Reproduced with permission [52]. Copyright 2022, Wiley.

Wang et al. [52] prepared PCBMA(1)-containing polymer brushes by a multistep synthesis that started with RAFT polymerization of glycidyl methacrylate (GMA) and transformation of glycidyl groups in PGMA into azide groups for “click” reactions (Figure 5) (Table 3, entry 7). The copolymers obtained (BCPB-F) containing grafted PCBMA(1) and fluorinated PFHEA segments had molar masses of ~11.9 kDa and assembled into cylindrical nanoparticles (~90 nm length, ~18 nm diameter) that remained stable in water, PBS, and PBS with 10% FBS for at least 28 days. They showed negligible cytotoxicity up to 200 μg/mL across bEnd.3, HT22, and CTX-TNA2 cell lines. Flow cytometry revealed that uptake of BCPB-F in bEnd.3 endothelial cells was 1.41-fold higher than BCPB-H (alkyl analog), 1.61-fold higher than non-fluorinated BCPB, and 4.0-fold higher than PEG-based ECPB. In vivo imaging confirmed that BCPB-F accumulated progressively in mouse brains, reaching a maximum at 72 h post-injection; the brain signal intensity was 1.32-fold higher than BCPB-H and ~1.9-fold higher than ECPB. DOX-loaded BCPB-F achieved the highest brain DOX concentration of 0.50 ± 0.15% ID/g at 72 h, compared with 0.27 ± 0.05% for BCPB-H and 0.26 ± 0.02% for BCPB, while levels for PEG-based ECPB were too low to quantify.

Xiao et al. [53] developed versatile diblock copolymers poly(2,2,3,4,4,4-hexafluorobutyl methacrylate)-b-poly(carboxybetaine methacrylate) (PFBMA-b-PCBMA(1)) via RAFT polymerization (Table 3, entry 15). Fluorine-free copolymer poly(butyl methacrylate) (PBMA-b-PCBMA(1)) was similarly synthesized. PFBMA-b-PCBMA(1) and PBMA-b-PCBMA(1) self-assembled in PBS into spherical nanoparticles of 110 and 145 nm in diameter, respectively. The PFBMA-b-PCBMA(1) copolymer had lower CAC than those of PBMA(1)-b-PCBMA. Both PFBMA-b-PCBMA(1) and PBMA-b-PCBMA(1) nanoparticles exhibited positive surface charge (~20 mV) due to significant protonation of the carboxyl groups. PFBMA-b-PCBMA(1) and PBMA-b-PCBMA(1) aggregates served as carriers of ciprofloxacin (CIP). The CIP-loaded PFBMA-b-PCBMA(1) had a smaller diameter (145 nm) and higher loading capacity (9.2%) than the CIP-loaded PBMA-b-PCBMA(1) aggregates (190 nm and 5.1%). The release of CIP from PFBMA-b-PCBMA(1) triggered by lipase led to 80% drug release, while without lipase it was under 15%. The nanoparticles exhibited long circulation, high bioaccumulation at infection sites, improved wound healing, and minimal tissue damage.

Yokota et al. [19] synthesized dual zwitterionic block copolymers of 2-(methacryloyloxy)ethyl phosphorylcholine and CBMA(2) (PMPC-b-PCBMA(2)) via RAFT (Table 3, entry 17). Polyion complex micelles were formed at pH 3 by mixing cationic PMPC-b-PCBMA(2) with anionic PMPC-b-poly(3-sulfopropyl methacrylate potassium salt), PMPC-b-PMPS block copolymers, driven by electrostatic interactions between PCBMA(2) and PMPS blocks. Increasing pH above 4 or adding NaCl (≥0.2 M) dissociated the micelles due to deprotonation of PCBMA(2) and electrostatic screening.

Li et al. [54] obtained a triblock copolymer consisting of PCBMA(2) side blocks and a central hydrophobic poly(propylene oxide) (PPO) segment [PCBMA(2)-b-PPO-b-PCBMA(2)] via ATRP in methanol (Table 3, entry 5). The PCBMA-based triblock copolymers were applied to modify various hydrophobic surfaces, including PDMS, where the triblock copolymer formed stable and dense films. On PCBMA_40_-PPO_48_-PCBMA_40_-coated PDMS, fibrinogen adsorption was reduced to ~3% of the uncoated surface, with SPR measurements confirming <2 ng/cm^2^ for single proteins and only 5.2 ng/cm^2^ for undiluted plasma. After EDC/NHS activation, the copolymer-coated surfaces enabled covalent immobilization of antibodies at 70.6 ng/cm^2^, enabling subsequent antigen detection at 58.2 ng/cm^2^, while maintaining antifouling performance. Residual activated groups reverted to zwitterionic form, keeping nonspecific adsorption at ~5% of uncoated PDMS even after biomolecule functionalization.

Carr et al. [55] synthesized copolymers using CBMA-EE monomers with different degrees of nitrogen atom substitution for efficient and biocompatible gene delivery. Monomers of tertiary and quaternary amine CBMA-EE were copolymerized through free radical polymerization with APS as the initiator in water at pH 5 (Table 2, entry 19). The synthesized copolymers formed DNA complexes as nanoparticles, ranging in size from 81 to 131 nm and slightly positive zeta potentials, which are ideal for cellular uptake. The hydrolysis of these PCBMA-EE copolymers facilitated DNA release and conversion of PCBMA-EE into zwitterionic PCBMA(1), significantly reducing cytotoxicity. Optimized 3:1 tertiary/quaternary hydrolyzable copolymers demonstrated transfection efficiencies up to 20 times higher than branched PEI, while also maintaining significantly higher cell viability. In contrast, copolymers containing non-hydrolyzable quaternary ammonium monomers also condensed DNA into nanoparticles of elevated cytotoxicity with slightly larger sizes ranging from 111 to 357 nm.

Li et al. [56] synthesized block copolymers with a main chain consisting of poly(acrylic acid) and poly(lactic acid). ATRP initiation sites were introduced to PAA through amidation, followed by ATRP of tBuCBMA and subsequent deprotection to PCBMA(1) (Table 2, entry 16). Copolymers provided with a chelator (DOTA) and tyramine residue for labeling with Cu-64 atoms were employed to produce degradable shell-crosslinked polymer nanoparticles whose pharmacokinetic profile was compared with PEG-functionalized counterparts. The study demonstrated that PCBMA(1)-grafted nanoparticles exhibited in vivo pharmacokinetics comparable to their PEG analogs. At 1 h post-injection, both PEGylated (2 kDa) and PCBMA-grafted (2 kDa) nanoparticles showed ~10% ID/g blood retention. However, by 4 h, PEGylated particles dropped to <5% ID/g, while PCBMA particles maintained >8% ID/g, and at 24 h they exhibited a distinct clearance profile with >16% ID/g excretion through kidneys. In contrast, PEG-coated nanoparticles were mainly cleared by the liver and spleen, showing higher MPS uptake. These results indicate that PCBMA provides comparable or improved pharmacokinetic behavior relative to PEG, avoiding hepatic uptake.

Kitano et al. [35] synthesized PCBMA(1) homopolymer (Table 2, entry 3), CBMA(1) copolymers with n-butyl methacrylate (BMA) (Table 3, entry 20), homopolymers of methyl methacrylate (PMMA), and n-butyl methacrylate (PBMA) for comparison, using free radical polymerization. The O–H stretching band of water incorporated in P(CBMA(1)-co-BMA) films was nearly identical to that of free water, indicating that the zwitterionic groups did not disturb the hydrogen-bonded network. In contrast, significant changes were observed for water incorporated in PMMA and PBMA films, reflecting disruption of bulk water structure. Quantitatively, the number of hydrogen bonds collapsed per monomer unit (Ncorr) was ~0 for P(CBMA(1)-co-BMA) (17.8 kDa, 45 mol% CBMA) and –0.27 for polyCBMA, values much smaller than those for ordinary polyelectrolytes (Ncorr ≈ 5–6) and comparable to PEG (0.7). Blood compatibility tests demonstrated that platelet adhesion to P(CBMA(1)-co-BMA) films was reduced compared to PMMA and PBMA surfaces. Relative to PBMA, normalized platelet adhesion dropped to a minimum at CBMA contents of 14–17 mol%, confirming a strong correlation between zwitterionic hydration and suppression of platelet adhesion. At higher CBMA content, platelet adhesion slightly increased due to surface roughness effects but remained significantly lower than for PMMA or PBMA films.

Despite these promising results, synthesis of PCBMA copolymers relies on controlled radical polymerizations and complex block designs, which might increase cost and complicate large-scale production. While copolymer assemblies have shown strong antifouling and drug-loading capacities in vitro and in small animal models, data on long-term stability, degradation, and reproducibility under physiological conditions are still limited. Addressing these challenges will be necessary before CBMA copolymers can realistically complement or replace PEG in clinical formulations.

**Table 3:** Copolymers of PCBMA and their characteristics.

Entry	Copolymers	Method	Synthesis of PCBMA Block	Yield	M_n_ [kDa]	Size of Aggregates [nm]	Loaded Drug	DLE, DLC	Size after Loading [nm]	Ref.
1	PLGA-b-PCBMA(1)	ATRP and Click	CuBr/HMTETA, DMF, and TFA deprotection	-	-	150	DOX	5% 1%	138.5	Cao et al. [26]
2	PDMNBMA-b-PCBMA(2)	ATRP	PDMAEMA-Br, CuBr/bpy, and CBMA(2)/MeOH		~9	-	BSA complexation	DLE 85.6, 92.7, 96.1%	229, 172, 142 nm	Jin et al. [48]
3	PCBMA(2)-b-p(ADA-TMDP)		CuBr/HMTETA, MeOH/DMF	-	15.3	69	DOX	35%, 14%	77	Ma et al. [37]
4	PCBMA-EE-b-PCBMA(2)		PCBMAEE_50_ macroinitiator, CBMA(2), and CuBr/PMDETA in methanol	-	14.5, 15.0, 15.6, 18.3		Plasmid DNA	-	150–200, 130–180, 93, (80–90 for N/P = 10/1)	Zhang et al. [36]
5	PCBMA(2)-PPO-PCBMA(2)		Br-PPO-Br macroinitiator, CBMA(2), and CuBr/bpy in methanol	-	19	-	-	-	-	Li et al. [54]
6	Dextran-g-[PDMAEMA-b-PCBMA(2)]		Dextran-g-PDMAEMA-macroinitiator, CBMA(2), and CuBr/PMDETA in MeOH/water (2/3)	-	82, 91, 104	-	Plasmid DNA		100~120 (N/P = 10/1)	Xiu et al. [49]
7	PGA-g-(PGMA/PHTE-b-PCBMA(1)-b-PFHEMA)	RAFT + ATRP + Click	t-BuCBMA and PMDETA in DMF	-	11.85	~20	DOX	-	~20	Wang et al. [52]
8	PCBMA(1)-b-PEMAAB	RAFT	CPDB/ACVA, CBMA(1), and DMF/H_2_O, 70 °C, 2 h	-	20, 22, 30	120–180, 30–40 after UV	-	-	-	Shrivastava et al. [43]
9	PCBMA(1)-b-PEHA		CPADB/ACVA, CBMA(1), and DMF/H_2_O(4/1), 70 °C, 2 h	24–40%	~19, ~25	6–12 layer thickness	-	-	-	Matsuoka et al. [44]
10	[P(n-BA)]-b-PCBMA(1)]		PCBMA(1)-macroCTA, nBA, and AIBN in MeOH, 70 °C	-	11.9, 25.2, 33.6, 64.5	64 and 79 in water, 65 and 86 in 1 M NaCl	-	-	-	Murugaboopathy et al. [45]
11	PCBMA(1)-b-PHPMA		PCBMA(1)-macroCTA, HPMA, and ACVA in water, 70 °C	99%	~45	34.5	-	-	-	Ning et al. [31]
12	PCBMA(2)-b-PCL-b-PCBMA(2)		CPADB-SS-PCL-SS-CPADB macro-CTA, CBMA(2), and AIBN in THF/saturated saltwater (1:1)	45%	~6	102	DOX	41%, 15%	124	Jiang et al. [51]
13	PCBMA(1)-b-PSBMA		CBMA(1)-macroCTA, SBMA, and VA-044 in H_2_O	43–70%	64.6, 59.0, 96.5, 85.8, 99.2, 82.3, 75.0	40.9, 31.9, 62.9, 39.7, 54.6, 51.1, 26.4 at 25 °C	-	-		Lim et al. [32]
14	PCBMA(1)-b-PSBMA, PSBMA-b-PCMA(1)-b-PSBMA		PCBMA(1) or PSBMA-macroCTA, PETTC, and VA-044 in H_2_O/TFE (8/2)	-	25.7, 31.2, 68.3, 40.1, 71.0		-	-	-	Lim et al. [21]
15	PFBMA-b-PCBMA(1), PBMA-b-PCBMA		PFBMA-macro-RAFT, t-Bu(CBMA), and AIBN	-	7.3, 5	110	Ciprofloxacin		145	Xiao et al. [53]
16	PCBMA(1)-g-(PAA-b-PLA)		CBMA-tBu in DMF; amidation onto PAA-b-PLA; and deprotection with TFA	-	~20, ~35	29, 65	Labeled with Cu-64 by an embedded chelator and tyramine	-	-	Li et al. [56]
17	PMPC-b-PCBMA(2)		PMPC-macroCTA, CBMA(2), and ACVA in water/methanol mixture	86.6%	23.5	18.9 at pH 3, 5–9 at pH >4	-	-	-	Yokota et al. [19]
18	P(NIPAM-co-CBMA(2))	Free radical	CBMA(2), NIPAM, and APS/TEMED in water	-	42.5, 44.8	115–135 at 37 °C, ~90 upon cooling down	-	-	-	Ζhao et al. [50]
19	2°, 3°, 4° CBMA–EE random copolymers		2°, 3°, and 4° CBMA–EE and APS in water (pH 5)	-	~30–35				81–352 (N/P = 40)	Carr et al. [55]
20	P(CBMA(1)-co-MBA)		CBMA(1), BMA, AIBN, and 2-mercaptoethanol in ethanol	-	11.4, 17.8, 347, 429, 163	-	-	-	-	Kitano et al. [35]

### 3.3. Star-Like (Co)Polymers of Carboxybetaine Methacrylate

The synthesis and study of star-like polymers containing CBMA are rather rare. Lin et al. [57,58] synthesized star-shaped PCBMA(2) polymers based on a β-cyclodextrin (β-CD) ATRP initiator, providing roughly six initiation sites for arm extension. The incorporation of RhB-HEMA introduced fluorescence into the polymer, allowing for tracking in biological studies. The hydrodynamic size of the stars in PBS ranged from 3.0 to 11.9 nm, increasing with molar mass. PSBMA stars were synthesized for comparison following the same ATRP. The study showed that the circulation half-life (t½) of PCBMA(2) stars in mice increased with the molar mass of the polymers, reaching 39.1 h for the largest (123 kDa) stars, compared with <10 h for PSBMA stars of similar size. After 6 h, 80% of PCBMA stars remained in circulation versus only 42% for PSBMA, and after 24 h, 55% of PCBMA remained compared to <10% for PSBMA, confirming the superior hydration and antifouling effect of PCBMA. Repeated injections did not induce an immune response; moreover, serum biochemistry and organ histopathology showed no significant differences between treated and control mice, indicating excellent in vivo biocompatibility. In vitro, the star-shaped polymers exhibited low cellular uptake, minimal macrophage internalization, and no detectable hemolysis at concentrations of up to 5 mg/mL. High cell viability (>90%) was observed for RAW 264.7 and HUVECs even at 2 mg/mL. PCBMA stars resisted nonspecific protein adsorption to a similar degree to PSBMA stars (<5% of TCPS control), but their longer circulation time in vivo showed a significant advantage of PCBMA over PSBMA as a blood-compatible stealth polymer.

**Figure 6:**
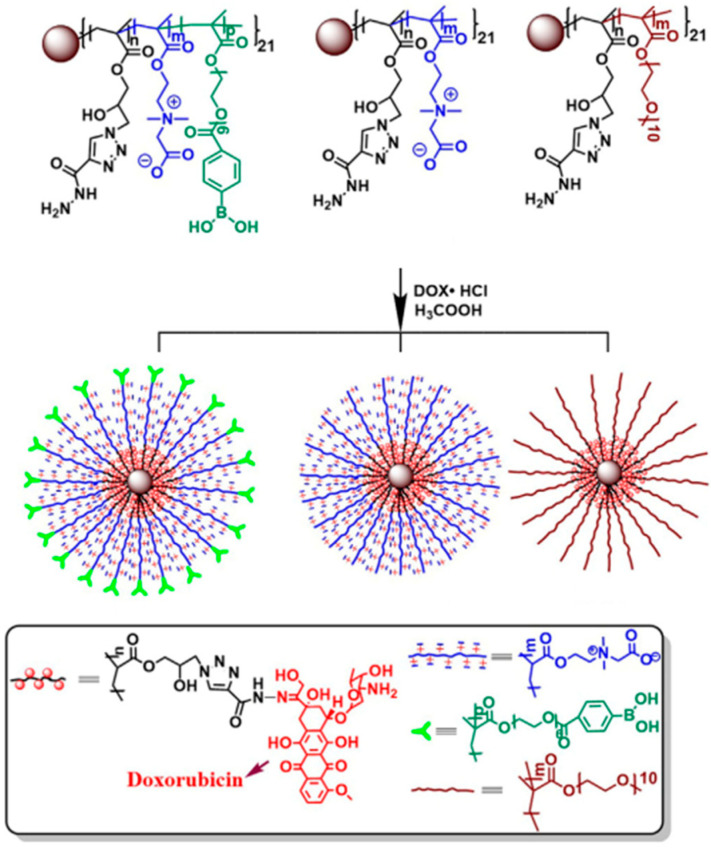
Stars with β-CD core and arm composed of CBMA, OEGMA-Bpin, or PEGMA and star conjugation with DOX. Reproduced with permission [59]. Copyright 2016, Elsevier.

Zhang et al. [59] synthesized star copolymers, containing 21-arm PCBMA(1)-b-PGMA via ATRP (Figure 6). Different star compositions were achieved by varying the amounts of PGMA and t-BuCBMA or by replacing t-BuCBMA with PEG. Another variation included an OEGMA block with terminal polyether side chains modified with 4-carboxyphenylboronic acid. Further functionalization involved converting the epoxy groups in the PGMA block into azido groups, allowing the conjugation of doxorubicin via pH-sensitive acylhydrazone linkages. The procedure is shown on Figure 6.

For all star polymers the surface charge was neutral at physiological pH and increased in acidic conditions. Controlled drug release was achieved in the acidic tumor microenvironment due to acylhydrazone linkages, with only ~25–30% of DOX released at pH 7.4 over 48 h, compared to 55–65% at pH 6.0 and up to 80% at pH 5.0. In vivo pharmacokinetics showed that PCBMA-based stars had elimination half-lives of 53–61 h, which were longer than PEG-based stars (~31 h) and earlier six-arm PCBMA stars (26–39 h). Biodistribution studies revealed that PCBMA-based stars delivered up to 7.0% ID/g of DOX to tumors, compared to ~4.2% for PEG-based stars and ~1% for free DOX. The therapeutic efficacy was increased for PCBMA-based stars, as tumor growth inhibition reached 88–91%, compared to ~83% for PEG stars and only 48.5% for free DOX, with significantly prolonged survival in the PCBMA groups. These results confirmed that PCBMA stars provide stronger hydration, longer circulation, and higher tumor accumulation than PEG analogs, making them particularly promising for nanomedicine design.

PCBMA star copolymers show superior circulation and immune-evasive properties compared to PEG and PSBMA stars, but important challenges remain. Their preparation often requires multi-arm initiators and precise control of polymerization, which complicates reproducibility and scalability. Existing pharmacokinetic studies have been limited to rodent models and the long-term biocompatibility, degradation behavior, and safety of high-molecular-weight star constructs remain underexplored. These factors currently limit their direct translation to clinical applications.

## 4. Applications of Poly(Carboxybetaine Methacrylate) in Biological Systems

### 4.1. Poly(Carboxybetaine Methacrylate) Conjugates with Biological Molecules

PCBMA is often conjugated with biological molecules or drugs to improve their biocompatibility and stability in biological systems. These conjugates benefit from the zwitterionic nature of PCBMA, which reduces immune responses and enhances the targeted delivery of therapeutic agents.

Keefe et al. [38] synthesized PCBMA(1) from t-BuCBMA for conjugation with chymotrypsin (CT) (Table 2, entry 7). Resistance to nonspecific protein absorption and CT binding affinity and stability were compared with PEG-CT analogs. Higher degrees of PEGylation progressively weakened binding, whereas PCBMA conjugates showed the opposite trend, with K_m_ decreasing as more chains were attached. Stability assays showed that PCBMA had increased protection under stress: in 5 M urea, PCBMA–CT conjugates retained ~80–100% activity compared to much lower residual activity for PEG–CT; under thermal stress at 55 °C, PCBMA conjugates maintained >70% activity compared to ~40–50% for PEG. Unlike PEG, which sterically hinders binding and weakens hydrophobic interactions, PCBMA’s superhydrophilicity reorganizes the local hydration shell to strengthen protein–substrate binding. As a result, PCBMA–CT conjugates maintained or improved activity relative to both free CT and PEG–CT, while also displaying enhanced stability to heat and destabilizers such as urea.

**Figure 7:**
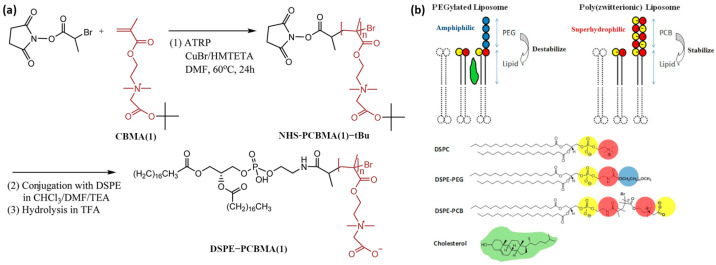
(**a**) The route of conjugate synthesis of PCBMA(1) with DSPE phospholipids; (**b**) schematic representation of DSPC/DSPE-PCBMA(1) hybrid liposome formation. Reproduced with permission [39]. Copyright 2012, American Chemical Society.

Cao et al. [39] prepared linear PCBMA(1) via ATRP for conjugation with lipids to improve the stability of the resulting liposomes (Figure 7a) (Table 2, entry 8). The obtained DSPE-PCBMA conjugates were mixed with DSPC lipids in PBS to form hybrid liposomes with mean diameters of 84–140 nm and zeta potentials of −32 to −48 mV, depending on composition and PCBMA chain length. For comparison, PEGylated analogs ranged from 92 to 160 nm with lower surface charges (−13 to −33 mV). Unlike PEGylated liposomes, which required cholesterol for long-term stability, PCBMA liposomes remained stable in PBS for >100 h at 37 °C and for over six months at 4 °C, which is consistent with the DSC data that showed a slight decrease in DSPC T_m_ (−1 °C) due to enhanced hydration, in contrast to PEG which increased T_m_ (+1 °C).

Carboxyfluorescein-loaded PCBMA liposomes exhibited excellent retention, comparable to cholesterol-stabilized DOXIL, while PEGylated liposomes without cholesterol leaked significantly. Circulation studies in rats showed that PCBMA liposomes displayed half-lives of ~20–30 h, similar to PEGylated liposomes, both much longer than unmodified DSPC (<5 h). When loaded with doxorubicin, the PCBMA liposomes (15 mg/kg DOX) delayed tumor growth more effectively than free DOX and achieved complete tumor elimination in four of five mice by day 60, with cures occurring ~6 days earlier than with DOXIL. All cured mice remained tumor-free during 90 days of observation, highlighting the therapeutic potential of PCBMA-based formulations.

**Figure 8:**
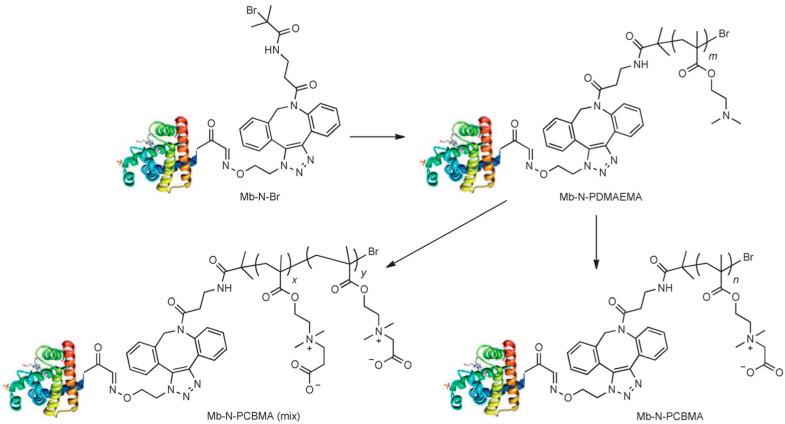
Synthetic route of Mb-N-PCBMA conjugates via ATRP and chemical modifications [60]. Copyright 2015, Wiley.

Bhattacharjee et al. [60] created site-specific protein conjugates of myoglobin and PCBMA (Figure 8): one by treating the amine groups with iodoacetic acid, which led to Mb-N-PCBMA(1) (molar mass 575 kDa), and one with 3-iodopropionic acid and iodoacetic acid, which resulted in the formation of a PCBMA(1) and PCBMA(2) conjugate mixture (Mb-N-PCBMA_mix_) of 600 kDa. A longer plasma circulation time of Mb-N-PCBMA(1) compared to Mb-N-PCBMA_(mix)_ conjugates was observed, highlighting how the spacer length between charges influences pK_a_ and dipole moment. Pharmacokinetic analysis showed that Mb-N-PCBMA(1) achieved an elimination half-life of 17 h and the highest plasma exposure (AUC 1.82 × 10^3^ h·%blood·mL^−1^), compared to 10 h and an AUC 9.89 × 10^2^ for Mb-N-PCBMA_(mix)_. Both conjugates outperformed both native Mb (3.1 h) and a cationic Mb-N–PDMAEMA precursor (3.2 h), while Mb-N-PCBMA(1) also exhibited longer circulation than the Mb-N–POEGMA PEG-like conjugate (13 h, AUC 1.44 × 10^3^). These results confirmed that PCBMA provided superior plasma retention relative to PEG analogs, with charge spacing playing a critical role in modulating in vivo behavior.

PCBMA(2)–curcumin conjugates for Aβ_42_ fibrillation inhibition were prepared by Zhao et al. [40] via ATRP polymerization in H_2_O/DMF (Table 2, entry 9). Above CAC, conjugates in PBS formed nanoparticles of 190, 142, and 122 nm. PCBMA(2)–curcumin conjugates self-assembled into nanogels (120–190 nm) with a solubility hundreds of times higher than free curcumin (0.27 μg/mL). Among them, PCBMA(2)-Cur (DS = 1.97) exhibited the strongest effect, inhibiting Aβ_42_ fibrillation by 47% at 5 μM, while free curcumin at the same concentration achieved only 15% inhibition; at 25 μM, PCBMA(2)-Cur reduced ThT fluorescence to 9% of the control, versus 60% for free curcumin. In SH-SY5Y cells, PCBMA(2)-Cur increased viability to 91% at 5 μM, compared to 76% with free curcumin, and protected against Aβ42-induced toxicity with cell viabilities of 73% at 5 μM, 77% at 10 μM, and 82% at 25 μM, which is higher than the values for free curcumin or HA–curcumin conjugates. These results confirmed that PCBMA conjugation not only enhanced curcumin’s solubility and colloidal stability but also amplified its inhibitory potency against Aβ aggregation and cytotoxicity.

**Figure 9:**
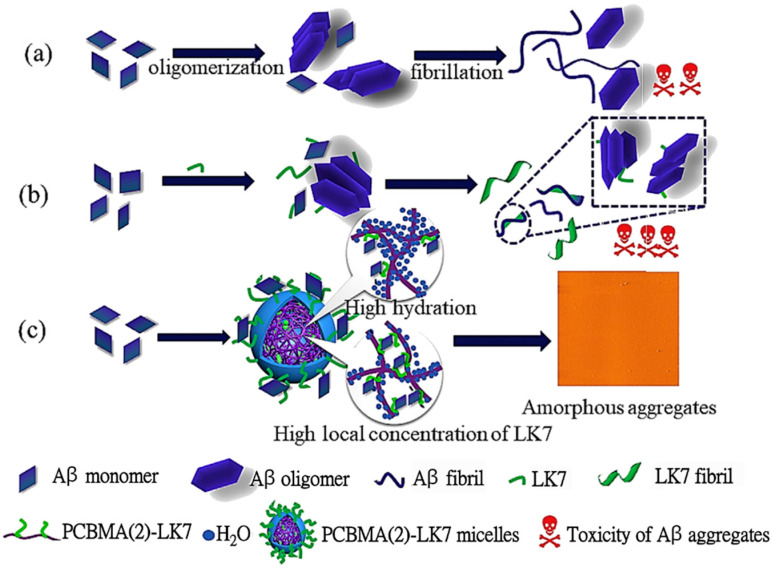
Schematic representation of (**a**) Aβ fibril formation, (**b**) the influence of LK7, and (**c**) the action of LK7@PCBMA conjugates, where the combined roles of PCBMA hydration and the elevated local concentration of LK7 within micelles contribute to the suppression of Aβ fibrillogenesis. Reproduced with permission [41]. Copyright 2019, Elsevier.

PCBMA(2) conjugates with Ac-LVFFARK-NH_2_ (LK7) peptide were prepared by Zhao et al. [41] via ATRP in H_2_O/DMF (Table 2, entry 10). The conjugation of LK7 to PCBMA(2) eliminated the self-aggregation of LK7 and led to the formation of amphiphilic nanoparticles with hydrophilic PCBMA(2) shell and a hydrophobic LK7 core, as shown in Figure 9. Depending on the degree of conjugation, the nanoparticles’ diameter ranged from 220 to 330 nm in PBS. PCBMA(2)-LK7 conjugates exhibited improved inhibitory effects on Aβ_42_ fibrillogenesis compared to free LK7, as shown by significant reduction in β-sheet formation. Circular dichroism measurements showed that free Aβ_42_ aggregates contained ~44.5% β-sheet, while the presence of PCBMA(2)-LK7 conjugates reduced this to 33.7% at 0.05 μM, 30.3% at 0.2 μM, and as low as 21.7% at 1 μM, compared to ~40–44% even with 25 μM free LK7. This remarkable improvement stemmed from the high local concentration of LK7 in the nanoparticle core and the stabilization provided by the highly hydrated PCBMA(2) shell. Cytotoxicity assays further confirmed the advantage: SH-SY5Y cells exposed to Aβ_42_ aggregates maintained only ~66% viability, which fell to ~51% with free LK7, but was restored to 97% with 0.2 μM PCBMA(2)-LK7 conjugates. These results demonstrated that PCBMA conjugation not only enhanced antifibrillation activity but also dramatically reduced the toxicity of LK7.

Baker et al. [42] studied lysozyme–polymer conjugates. Conjugates of zwitterionic PCBMA(2) (Table 2, entry 11) and neutral POEGMA were synthesized using grafting from ATRP with high grafting density and varied polymer chain lengths. For Lyz-PCBMA(2) conjugates, the hydrodynamic diameters in PBS increased from 7.9 nm (DP 18) to 16.8 nm (DP 91) with increasing chain length. Similarly, the hydrodynamic diameters for Lyz-POEGMA conjugates ranged from 9.2 nm (DP 25) to 26.2 nm (DP 164). The solubility of lysozyme–polymer conjugates in ammonium sulfate solutions varied with polymer type and length. Lyz-PCBMA(2) conjugates remained fully soluble even at 100% saturation (4.1 M), regardless of polymer length. Their hydrodynamic diameter increased with ammonium sulfate concentration, without precipitation. Conjugates remained stable in fully saturated ammonium sulfate concentrations for up to 2.5 months, independent of PCBMA(2) molar mass. In contrast, Lyz-POEGMA conjugates exhibited length-dependent precipitation.

Peng et al. [46] explored PCBMA’s ability to form polyplexes with RNA. PCBMA homopolymers were synthesized from CBMA(2) in methanol, using 2-cyanopropan-2-yl benzodithioate as the RAFT CTA and AIBN as the initiator. PCBMA polyplexes of 6.49 kDa and 11.65 kDa were obtained with a yield of 75.0% (Table 2, entry 19). The PCBMA(2)-siRNA polyplexes formed spherical nanostructures with the diameter decreasing with increasing N/P ratios. The mean diameter of longer PCBMA(2)-siRNA at an N/P ratio of 30 was 128.2 nm. The surface charge of the PCBMA(2) polyplexes remained nearly neutral. The longer PCBMA(2) resulted in comparable siRNA loading capability with PEI. The PCBMA(2)’s ability to load siRNA increased with decreasing pH. The PCBMA(2)-siRNA polyplexes were able to reduce the level of PLK1 protein in HeLa cells, promoting apoptosis but maintaining higher cell viability across various N/P ratios. The PCBMA(2)-siRNA polyplexes showed enhanced cellular uptake in tumor environments and could efficiently escape from endosomes and lysosomes into the cytoplasm, ensuring effective siRNA delivery.

Jazani et al. [47] investigated the use of photo-RAFT polymerization to create PCBMA–chymotrypsin hybrids. They used sodium pyruvate (SP) and its derivatives to generate radicals under UV light and allowed polymerization to occur in water at room temperature, even in the presence of oxygen. CBMA(2) homopolymerization was carried out in a water/DMSO mixture using CPADB as the RAFT chain transfer agent (CTA), resulting in a 31.6 kDa polymer (Table 1, entry 20a). To synthesize the enzyme–polymer hybrid, CT was first modified by attaching the RAFT CTA and then used to initiate polymerization in water, yielding two PCBMA(2)–enzyme conjugates with molar masses of 80.2 and 92.9 kDa (Table 1, entry 20b). The polymerization was well-controlled, producing polymers with high monomer conversion and low dispersity. The PCBMA(2)-CT hybrids retained their enzymatic activity and the presence of PCBMA improved their antifouling properties.

Conjugation of biomacromolecules with PCBMA resulted in great stability and protection of enzymatic/peptide activity. Conjugation strategies often require multi-step chemistries and precise control over substitution levels, which complicates reproducibility and scaling. Most studies compare performance only to PEG or PEI in vitro and in small animal models, with little data available on long-term safety, biodistribution, or metabolic clearance of PCBMA conjugates. Further systematic evaluations will be necessary before these systems can advance toward clinical translation.

### 4.2. Nanogels for Drug Delivery

PCBMA-based nanogels demonstrate significant potential in enhancing the efficacy and safety of therapeutic and diagnostic modalities. PCBMA nanogels can evade protein adsorption and immune system recognition, resulting in prolonged circulation times in the bloodstream. Responding to specific environmental triggers, such as changes in pH or redox conditions, they can enable controlled and targeted drug release.

In the earliest studies, Cheng et al. [61] developed multifunctional PCBMA-based nanogels via inverse microemulsion free radical polymerization, using CBMA(2). The resultant nanogels had a size ranging from 99 to 117 nm, depending on the crosslinker content (1.5–5% MBAA). Nanogels exhibited good stability in 100% FBS, maintaining their original size over 18 h and showed minimal cytotoxicity against HUVECs. Fluorescein isothiocyanate-labeled dextran was encapsulated, demonstrating controlled release over 18 days. Cyclo[Arg-Gly-Asp-D-Tyr-Lys] (cRGD) ligands were further attached to the nanogels, significantly enhancing cellular uptake.

**Figure 10:**
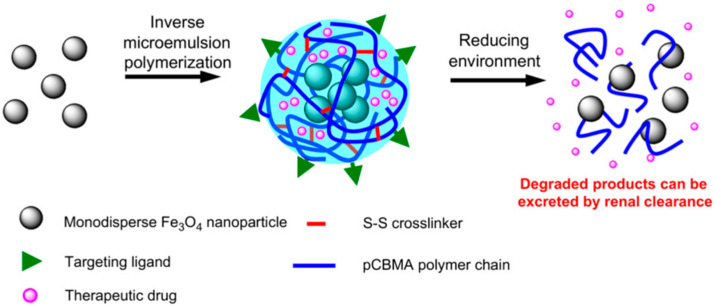
PCBMA(2) MNP-loaded nanogels crosslinked with L-cystine S-S crosslinker and their disassembly in reducing environments. Reproduced with permission [62]. Copyright 2011, Elsevier.

Zhang et al. [62] developed multifunctional and degradable PCBMA(2) nanogels with incorporated Fe_3_O_4_ monodisperse magnetic nanoparticles (MNPs) and fluorescein-labeled dextran (FITC–dextran) for enhancing MRI imaging (Figure 10). Synthesis was conducted via free radical inverse microemulsion polymerization of CBMA(2). Nanogels containing MNPs were obtained by formation of microemulsions using a water/hexane system. The PCBMA(2) MNP/FITC–dextran spherical nanogels had a hydrodynamic size of approximately 110 nm in PBS. They maintained their size for over six months in PBS and can be lyophilized to dry powder and re-dispersed in PBS without changing their size. The nanogels showed enhanced MRI performance compared to free-MNPs in both macrophage cells and HUVECs. The conjugation of RGD peptide to PCBMA(2)-based nanogels led to their significantly higher uptake by HUVECs and low uptake by macrophages. Fluorescein-labeled dextran was efficiently released in the presence of DTT, which degraded the disulfide bonds of the L-cystine bisacrylamide incorporated units in the PCBMA(2)-based nanogels. A total of 80% of the encapsulated FITC–dextran was released over 48 h. Without DTT, the release was only 3%. The degradation products of the nanogels, which include small MNPs and PCBMA(2) polymer chains, can be removed from the body via renal clearance.

Zhang et al. [63] synthesized PCBMA nanogels through an inverse microemulsion polymerization method in hexane using CBMA(2). By adjusting crosslinking densities (2%, 5%, 10%, and 15%) and reactant content (40% or 46%), nanogels with varying stiffness were achieved. The PCBMA nanogels exhibited an average hydrodynamic size of approximately 120 nm and remained stable in PBS for over 60 days. Their ultralow fouling nature led to negligible uptake of macrophages compared to uncoated gold nanoparticles. In vivo studies revealed that softer PCBMA nanogels had significantly longer circulation half-lives compared to stiffer nanogels. While liver uptake was consistent across all nanogels, the deformability of softer nanogels allowed them to pass more effectively through splenic filtration slits and reduced splenic accumulation.

Lin et al. [64] prepared P(CBMA(2)-co-2-(methacryloyloxy) ethyl lipoate) P(CBMA-co-MAEL) via free radical polymerization in methanol. In methanol, P(CBMA(2)-co-MAEL) copolymers exist as unimers with diameters ranging from 6.2 to 7.2 nm. In water, nanogel size ranges from 23 nm to 183 nm were obtained. DOX loading leads to a significant increase in size up to 253 nm. The drug DLC and EE improved with increasing MAEL content. The DOX-loaded nanogels exhibited lower cytotoxicity compared to free DOX. The nanogels showed effective DOX release in the presence of intracellular glutathione or DTT. The carriers remained stable over 3 days in either PBS solution containing fibrinogen (1 mg/mL) or 50% FBS, demonstrating the nanogels’ resistance to protein adsorption and lack of aggregation in biological fluids.

Ding et al. [65] prepared PCBMA(2)-based nanogels composed of a poly(2-(diisopropylamino)ethyl methacrylate) (PDPA) crosslinked core and a PCBMA(2) shell. PDPA-PEG nanoparticles were also created for comparison. The PDPA-PCBMA(2) nanogels had a hydrodynamic diameter of approximately 237 nm and a slightly negative zeta potential. The PDPA core undergoes protonation at lower pH values, resulting in a significant increase in particle size due to intermolecular repulsion among positively charged PDPA segments. PDPA-PCBMA(2) nanoparticles functionalized with a targeting RGD molecule exhibited high specificity for U87 cells expressing α_v_β_3_ integrin, while showing low nonspecific interactions with HeLa and RAW 264.7 cells. The low-fouling property of PDPA-PCBMA nanogels was comparable to PDPA-PEG analogs, with <3% association with HeLa cells and <10% with RAW 264.7 macrophages after 10 h at 100 µg/mL, in contrast to >15% and >45%, respectively, for uncoated PDPA nanogels.

Mai et al. [27] prepared PCBMA-based nanogels via reflux precipitation polymerization (RPP), without the use of surfactants. The size and uniformity of the PCBMA(1)-based nanogels can be controlled by adjusting either the ethanol or the crosslinker content during the synthesis. Diameters in PBS can range from 82 to 236 nm by decreasing the ethanol content during the synthesis, while the diameter can increase from 100 to 175 nm by decreasing the amount of BAC crosslinker. The nanogels were stable for at least 7 days in both PBS and BSA solutions. DOX-loaded PCBMA(1)-based nanogels showed high release rates in the presence of glutathione at different pH levels, due to the degradation of disulfide bonds of the crosslinker in the reductive environment.

The DOX-loaded PCBMA(1)-based nanogels exhibited prolonged circulation (t½ ~9.0 h with ~18–19% blood retention at 24 h, compared to 5.1/3.6 h and 5–11% for POEGMA nanogels), did not trigger IgM/IgG immune responses unlike POEGMA, and showed enhanced tumor targeting with intratumor accumulation of 11.0% ID/g at 48 h versus 3.0% for POEGMA. Tumor growth inhibition was 68.7% for PCBMA-DOX compared to 48.8% for POEGMA-DOX and 33.5% for free DOX, alongside reduced systemic toxicity and improved safety.

PCBMA-based nanogels show strong antifouling and therapeutic performance, although several challenges remain. Their architecture is often obtained through multi-component or stimuli-sensitive designs that add synthetic complexity and may hinder large-scale reproducibility. Stability and performance have been demonstrated mainly in small animal models, and it is not yet clear how these PCBMA-based nanogels behave under long-term physiological stress or in larger organisms. Detailed toxicology and regulatory data are still lacking, which currently limits the translational outlook for PCBMA nanogels compared to clinically established PEG formulations.

### 4.3. Hydrogels for Tissue Engineering

Being one of the first, Zhang et al. [66] prepared transparent PCBMA(2) hydrogels by combining CBMA(2) monomer with tetraethylene glycol dimethacrylate and initiating free radical polymerization using sodium metabisulfite and ammonium persulfate. This mixture was prepared in a solution of ethylene glycol, ethanol, and water in a 3:1:1 volume ratio. The toxicity of PCBMA(2) hydrogels was found to be very low with endotoxin values lower than 0.06 endotoxin units per mL (EU/mL). The authors also reported antifouling properties with high resistance to cell and protein adhesion.

Zhang et al. [67] investigated PCBMA(2) and P(CBMA(2)-co-HEMA) hydrogels synthesized via free radical polymerization. The resulting hydrogels were transparent, highly hydrated, and equilibrated in PBS before testing. PCBMA(2) hydrogels effectively minimized protein adsorption due to their super-hydrophilic properties, which made them highly resistant to bovine aortic endothelial cell (BAEC) adhesion in vitro, unlike PHEMA hydrogels. After 5 days of incubation, BAECs adhered strongly to TCPS and moderately to PHEMA, whereas no adhesion was observed on PCBMA surfaces. In vivo, after 1 week of subcutaneous implantation in mice, PHEMA disks were densely covered with cells, while P(CBMA-co-HEMA) showed patchy cell attachment and polyCBMA hydrogels exhibited only sparse coverage. After 4 weeks, capsule vascularity analysis revealed that blood vessel density around PHEMA implants was 14 vessels/mm^2^, compared to 24 vessels/mm^2^ for P(CBMA-co-HEMA) and 29 vessels/mm^2^ for PCBMA, indicating a significant pro-angiogenic effect of zwitterionic hydrogels. Capsule thickness and FBGC density were similar across groups, confirming that the key difference was the improved antifouling and vascularization-promoting properties of PCBMA-based hydrogels compared to PHEMA.

Cheng et al. [30] developed hydrogels of CBMA-EE SA or CBMA(2) monomers by free radical polymerization. The PCBMA-EE SA hydrogel exhibited antimicrobial activity by inhibiting the growth of both Escherichia coli K12 and Staphylococcus epidermidis by 99.9% after 24 h at 37 °C. PCBMA(2) has not shown antibacterial properties. The PCBMA-EE SA hydrogel showed higher water content (96.57%) compared to the PCBMA(2) hydrogel (93.71%), which was attributed to its polyelectrolyte properties. Upon hydrolysis, the PCBMA-EE SA hydrogel transitioned to PCBMA(1), significantly reducing protein adsorption and bacterial adhesion. The PCBMA(2) hydrogel demonstrated excellent nonfouling properties but lacked antimicrobial activity.

Carr et al. [68] developed a range of nonfouling CBMA(2)-based hydrogels. In the first stage of work on hydrogels, carboxybetaine dimethacrylate (CBMAX-1) was used as a crosslinking agent, and hydrogels obtained in this way were compared with hydrogels based on traditionally used MBAA. A hydrogel prepared from CBMA(2) and crosslinked with CBMAX demonstrated significantly lower cell adhesion compared to MBAA-crosslinked hydrogels, which became more pronounced at higher crosslinker contents. The presence of CBMAX also led to significantly higher compressive moduli compared to MBAA-crosslinked hydrogels. In a follow-up study, Carr et al. [69] used a new crosslinker, CBMAX-2, containing two carbon atoms between a charged nitrogen atom and a carboxyl group. In addition to thermally obtained CBMA(2) hydrogels, similar hydrogels were also obtained by photopolymerization, leading to a significant improvement in network homogeneity and a wide range of compressive moduli from 0.5 to 90 MPa. In a follow-up study, Carr et al. [70] obtained hydrogels that exhibit varying gradient properties across the sample cross-section, which were named gradient hydrogels. CBMA(2) gradient hydrogels were prepared using a gradient-changing concentration of CBMAX-1/CBMAX-2 crosslinkers in continuous hydrogel formation. The obtained gradient nonfouling hydrogels were characterized by gradient-controlled compressive modulus, crosslinking density, and functionality depending on the hydrogel cross-section and depending on the concentration and CBMAX-1/CBMAX-2 crosslinker ratio used.

Yang et al. [71] synthesized PCBMA(2) hydrogels as coatings for glucose biosensors to improve performance in complex media like undiluted human blood serum. The PCBMA(2) hydrogels exhibited a high equilibrium water content of 91% (compared to 40% for PHEMA hydrogels) and showed a 90% reduction in cell adhesion compared to PHEMA. The PCBMA(2) hydrogels were applied to the glucose sensors by physical adsorption and chemical attachment to the platinum surface of the sensors. Physical adsorption involved directly coating the sensor tip with the hydrogel solution and curing it under UV light. Chemical attachment employed the covalent connection of PCBMA(2) hydrogel to the surface. Glucose sensors coated with PCBMA(2) hydrogels demonstrated excellent linearity, sensitivity, and stability in detecting glucose in wide range of its concentrations in PBS and human blood serum. The chemically attached PCBMA(2) hydrogels significantly outperformed physically adsorbed coatings, retaining sensitivity and linearity even in undiluted serum.

Beltrán-Osuna et al. [72] reported the synthesis of hydrogels prepared from CBMA(2) monomer using ATRP. PCBMA(2) was integrated into silica hydrogels using a two-step acid–base-catalyzed sol–gel process, resulting in materials with high surface areas. The incorporation of PCBMA(2) into silica hydrogels significantly enhanced their antifouling properties, achieving an 83% reduction in fibrinogen adsorption at 25 wt% PCBMA content. At low PCBMA concentrations (<20 wt%) the polymer uniformly integrated into the silica network, reducing particle size and increasing surface area by 91% compared to unmodified silica aerogels. At higher PCBMA concentrations (>33 wt%), polymer aggregation disrupted the silica structure, resulting in larger particle sizes and reduced network uniformity. These modifications contributed to optimal antifouling behavior at intermediate PCBMA levels (25 wt%). The study highlights the potential of these materials in applications such as drug delivery, filtration, and scaffolds, where high surface area and resistance to protein adsorption are critical.

Chien et al. [73] prepared biodegradable and functionalizable PCBMA(2) hydrogels via redox polymerization in PBS. Another hydrogel was prepared in a similar way, using an RGD-functionalized CBMA monomer. The hydrogels consisted of approximately 97% water and their diameter in the swollen state was twice that of the unswollen form, highlighting their remarkable swelling ability. NIH-3T3 fibroblasts cells were encapsulated within the PCBMA(2) and PCBMA(2)-RGD hydrogels. RGD-functionalized hydrogels supported better cell adhesion and proliferation compared to unmodified PCBMA(2) hydrogels. Cells recovered from the hydrogels maintained their morphology and proliferation.

Chien et al. [74] prepared PCBMA(2) hydrogels using a thiol–disulfide exchange reaction between a pyridyl disulfide-containing copolymer of PCBMA(2) and PCBMA-co-PDPMA and a thiol-functionalized crosslinker (PCBAA-DT). A 2% crosslinker and 7% PCBMA-co-PDPMA, which exhibited excellent gelation, mechanical stability, and biocompatibility, were chosen to encapsulate the cells in the hydrogel. The hydrogels supported the encapsulation of NIH-3T3 fibroblasts, MG63 osteoblast-like cells, and HepG2 hepatocarcinoma cells, maintaining over 90% viability for up to nine days under serum-free conditions. To enhance cell–matrix interactions, RGD-functionalized hydrogels were prepared by incorporating a cysteine-terminated RGD peptide during gelation. This significantly improved cell proliferation compared to non-functionalized hydrogels allowed for the recovery of encapsulated cells and maintained their morphology and proliferation rates.

Mi et al. [75] synthesized PCBMA(2), PCBMA-EE SA, and poly(2-(2-((2-(methacryloyloxy)ethyl)dimethylammonio)acetoxy)benzoate) (PCBSA) hydrogels. The CBMA-EE SA monomer was synthesized through a four-step reaction, involving the conjugation of salicylic acid (SA) to a carboxybetaine unit via a hydrolyzable ester bond. After polymerization, the hydrogels were hydrated. For PCBMA(2), the hydration occurred in either water or sodium salicylate solution. For PCBMA-EE SA and PCBSA, hydration was carried out under specific conditions (4 °C pH ~4) to stabilize the polymer and minimize premature hydrolysis. PCBSA demonstrated a sustained and controlled release profile of SA during hydrolysis, without requiring chemical modification of SA, ensuring the preservation of its bioactivity and converting PCBSA to PCBMA-EE. PCBSA exhibited minimal protein adsorption comparable to the highly nonfouling PCBMA(2), which was attributable to its zwitterionic nature and strong hydration. Resistance to bacterial adhesion was evaluated using Staphylococcus epidermidis, where PCBSA hydrogels showed significantly lower bacterial surface density than the cationic PCBMA-EE SA, maintaining a performance similar to PCBMA(2). PCBSA hydrogels achieved complete bacterial growth inhibition (>99%) against Staphylococcus epidermidis, combining bulk antimicrobial activity with a nonfouling surface. They also demonstrated high hydrophilicity, with a contact angle close to 0°.

Yang et al. [76] investigated lightly crosslinked PCBMA hydrogels for long-term glucose biosensors operating in whole blood. The CBMA(2) hydrogels were synthesized using CBMAX instead of conventional PEG-based crosslinkers. The hydrogel with 0.1% of crosslinker exhibited the highest equilibrium water content (94%) and the lowest nonspecific protein adsorption. This formulation allowed free diffusion of proteins and glucose within the hydrogel matrix without entrapment or adhesion. Hydrogels with higher crosslinker densities (1%, 10%, and 20%) showed reduced hydration and slightly increased protein interactions. Glucose oxidase (GOx) was covalently immobilized onto the hydrogels using sulfo-NHS/N-(3-dimethylaminopropyl)-N′-(ethylcarbodiimide hydrochloride) (EDC) chemistry, which reduced enzyme leaching and preserved bioactivity. The 0.1% crosslinked PCBMA hydrogel-coated sensors demonstrated exceptional sensitivity, linearity, and stability for detecting glucose concentrations ranging from 0.1 to 30 mM, outperforming commercial glucose sensors. The sensors maintained performance for up to 42 days in whole blood, with a detection limit of 0.1 mM and no need for calibration, which is a major advantage over conventional systems.

Zhang et al. [7] studied the in vivo performance of PCBMA(2) hydrogels crosslinked with CBMAX. After 1 week of implantation, histology revealed numerous inflammatory cells at the PHEMA–tissue interface but very few at the PCBMA interface. By 4 weeks and 3 months, all PHEMA samples were encapsulated by dense, avascular collagen with >90% collagen density at the interface, whereas PCBMA hydrogels showed no capsule formation, with collagen density remaining a uniform 30–40%, similar to a normal extracellular matrix. PCBMA hydrogels also promoted neovascularization, with significantly higher blood vessel density than PHEMA, and macrophage staining indicated a shift toward an anti-inflammatory, pro-healing phenotype at PCBMA implants. These findings confirmed that PCBMA hydrogels caused decreased inflammation and supported greater angiogenesis than PHEMA counterparts, even over 3 months of implantation.

Lin et al. [58] compared the in vivo and in vitro behavior of PCBMA(2) and PSBMA hydrogels. The hydrogels were prepared in an aqueous medium by radical polymerization of CBMA(2) or SBMA with TEGDMA and APS as the initiator. Although in vitro differences between the two hydrogels were minimal—both in terms of resistance to nonspecific protein adsorption and degree of internalization by cells—in vivo results showed a significant advantage of PCBMA(2) over PSBMA. The greatest difference was in circulation time, where PCBMA(2) showed an order of magnitude longer circulation time than PSBMA.

Chien et al. [77] developed an in situ-forming PCBMA(2) hydrogel specifically designed for tissue engineering. Gels from PMPC and PSBMA were also obtained in a similar manner for comparison. PCBMA(2) hydrogels reduced nonspecific protein adsorption and cell adhesion, minimizing immune responses and foreign body reactions in vivo after subcutaneous injection in murine models. They exhibited a compressive modulus of 23.6 ± 1.6 kPa, which was nearly 10-fold higher than PSBMA hydrogels (2.1 ± 0.5 kPa), while PMPC hydrogels were too soft to solidify. Both PCBMA and PSBMA hydrogels had high swelling capacities with ~95% water content, but only PCBMA maintained its original shape up to 3 weeks post-implantation, whereas PSBMA and PMPC dissolved within 2 days. Histology revealed minimal macrophage infiltration and thin capsules for PCBMA hydrogels, confirming their low-fouling and biocompatible nature. Moreover, PCBMA hydrogels were capable of encapsulating fibroblasts with ~90% cell viability, demonstrating their suitability as injectable, cell-supportive scaffolds.

Cai et al. [78] used powdered activated carbon hemoadsorbents by incorporation into PCBMA(2) hydrogels. The PCBMA(2)-PAC composite showed a high degree of swelling and mechanical strength. The stability of the PAC particles was significantly improved by hydrogel encapsulation. The composites were highly stable with negligible release of fine PAC particles during use. PCBMA(2)-PAC exhibited superior adsorption of bilirubin compared to PAC. The composite effectively resisted protein adsorption, even in 100% serum, and caused negligible hemolysis compared to pure PAC. The PCBMA(2)-PAC hydrogel ensured homogeneous adsorption and contributed to efficient mass transfer, enhancing the overall performance of the adsorbent.

Zhao et al. [23] synthesized hydrogels of CBMA(2), CBAA, and MPC using either MBAA or NDMCC as crosslinkers, APS as the radical initiator, and TEMED as the radical accelerator. PCBMA(2) hydrogels exhibited a significant amount of bound water (~5–6 wt%, Wb/Wtc ≈ 6–7), depicting stronger interactions with water molecules than PSBMA (~2–3 wt%) and PMPC (~1–2 wt%) hydrogels, though lower than PCBAA (9–15 wt%). They showed excellent antifouling behavior, with fibrinogen adsorption below 6.4% (versus 28.7% for PHEMA) and no cell adhesion. The water diffusion coefficient of PCBMA hydrogels was 8.2 × 10^−10^ m^2^/s, which was slightly lower than PCBAA (11.9 × 10^−10^ m^2^/s) and PMPC (10.9 × 10^−10^ m^2^/s), but still efficient in water transport. PCBMA hydrogels demonstrated the highest compressive modulus (34.3 kPa) and break stress (0.595 MPa) among the four zwitterionic hydrogels studied, confirming their improved mechanical strength, which was attributed to their distinct network structure.

Ruseva et al. [79] developed PCBMA(2) hydrogels for chronic wound dressings. All PCBMA(2) hydrogels exhibited extensive swelling behavior in acidic conditions, due to the protonation of carboxylic groups. With the increase in pH to alkaline values, resembling typical conditions of chronic wounds, the swelling decreases. In the presence of salt, hydrogel swelling ability increased. This was beneficial for absorbing wound exudate, which often contains salts. The hydrogels’ stability and robustness were maintained even after absorbing large amounts of fluid. The PCBMA(2) hydrogels were non-cytotoxic; human foreskin fibroblast cell viability remained above 75% without significant immune response. They inhibited biofilm formation by Staphylococcus aureus and reduced the presence of collagenase and myeloperoxidase by 30–40%, which is often elevated in chronic wounds.

Zhu et al. [80] prepared PCBMA(2) hydrogel with a pH indicator dye (phenol red) and two glucose-sensing enzymes, glucose oxidase and horseradish peroxidase, using CBMA(2). The PCBMA(2) matrix enhanced the stability and activity of the embedded enzymes. Enzymes catalytic efficiency was improved compared to their free forms. The hemolysis rate of the hydrogels was below the 5% threshold required for clinical applications. Red blood cell morphology remained intact and smooth. PCBMA(2) hydrogels led to faster healing in diabetic mice compared to the commercial DuoDerm dressing. By day 8, wounds treated with PCBMA hydrogels had contracted by ~73% in diabetic mice (vs. ~40–45% with DuoDerm) and ~88% in healthy mice (vs. ~60% with DuoDerm). By day 14, PCBMA hydrogels achieved complete closure in both groups, whereas DuoDerm-treated diabetic wounds still displayed lesions with an average epithelial gap of 1.57 mm. Histological analysis confirmed superior re-epithelialization and collagen regeneration in the PCBMA group, demonstrating the pro-healing advantage of PCBMA dressings.

**Figure 11:**
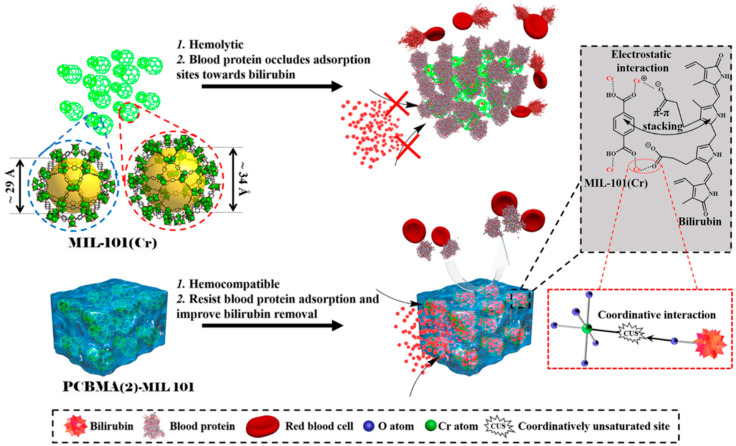
Schematic representation of MIL-101(Cr) and PCBMA-MIL101 hybrid hydrogels and their properties. Reproduced with permission [81]. Copyright 2020, American Chemical Society.

Li et al. [81] prepared a novel metal–organic framework (MOF)-based hemoadsorbent, PCBMA-MIL101, by encapsulating MIL-101(Cr), chromium-based crystals, with terephtalic acid ligand within a PCBMA hydrogel. This hybrid material was designed for hemoperfusion applications, specifically targeting bilirubin, a pathogenic toxin. PCBMA(2)-MIL101 hydrogels showed a high swelling ratio and mechanical strength. The incorporation of MIL-101 into PCBMA(2) prevented significant hemolysis observed with uncoated MIL-101(Cr). In biological media (BSA solution, 100% FBS), the hybrid hydrogels exhibited faster adsorption kinetics and higher bilirubin adsorption capacity than free MIL-101(Cr). PCBMA(2) was critical in maintaining bilirubin adsorption with a maximum capacity higher than many other reported hemoadsorbents. This was due to the large surface area and pore volume of MIL-101(Cr) and the efficient protection and permeability provided by the PCBMA(2) hydrogel (Figure 11).

He et al. [82] developed hydrogels using a multivinyl polycarboxybetaine polymer. The process started with the synthesis of a zwitterionic polycarboxybetaine macromonomer (PCBMA-OAA) through multiple steps, starting from a CBMA-OH monomer. First, the CBMA-OH monomer was polymerized in ultrapure water with ACVA as the initiator to form PCBMA-OH. Then, PCBMA-OH was modified by reacting it with acrylic anhydride in a weak alkaline solution, introducing multivinyl acrylate groups. This reaction resulted in the final macromonomer PCBMA-OAA. To create the hydrogel, a thiol–acrylate Michael addition reaction was performed, where PCBMA-OAA was crosslinked with DTT. The hydrogel demonstrated stable swelling behavior, reaching equilibrium within three days. The PCBMA-OAA hydrogel showed over 90% light transmittance and a refractive index similar to the natural vitreous body, which made the hydrogel suitable for use as a replacement for the ocular gel. The hydrogel has excellent antifouling properties. This ensures high cell viability and the rheological properties were similar to those of the natural vitreous body. The PCBMA-OAA hydrogel maintained retinal integrity and function over six months in vivo.

Ruseva et al. [83] focused on developing a smart interpenetrating polymer network (IPN) composed of PCBMA(2) and PSBMA. The IPN provided improved mechanical strength, compared to single PCBMA(2) and PSBMA networks, due to the mutual interlacing of the polymer chains. The storage modulus (G′) of the PCBMA(2)/PSBMA IPN was significantly higher than its loss modulus (G″), and higher than those of the single PCBMA(2) and PSBMA hydrogels. The swelling ratio of the IPNs increased almost twice as the temperature increased, due to PSBMA, which expands upon heating. In acidic conditions (pH < 5), IPNs expanded due to the protonation of the -COOH groups in PCBMA(2). Networks also expanded by increasing salt concentration due to the shielding effect of NaCl ions on the dipole–dipole interactions between the zwitterionic moieties. The PCBMA(2)/PSBMA IPNs effectively inhibited bacterial growth, demonstrating antibiofouling performance comparable to the neat PCBMA(2) hydrogel and better performance than the PSBMA hydrogel. The IPNs were non-cytotoxic, supporting cell proliferation and making it a suitable candidate for biomedical applications.

Qi et al. [84] obtained PCBMA(2)-based composite hydrogels of CBMA(2) in a mixture of well-dispersed Fe_3_O_4_ nanoparticles in water, Span 80, and Tween 80, with APS as an initiator, N,N′-methylenebisacrylamide (MBAA) as a crosslinker, and TEMED. The formed hydrogels were further complexated with lipase. The results showed enhanced pH and temperature tolerance of the immobilized lipase over a broader pH range and at higher temperatures compared to the free enzyme. The catalytic efficiency of the immobilized enzyme was increased by approximately 50%, as shown by a high molecule conversion/substrate affinity (K_cat_/K_m_) ratio. The PCBMA(2) hydrogels showed no impact on NIH 3T3 cells in comparison with naked Fe_3_O_4_ particles that caused a significant cell viability reduction of 50%.

Liu et al. [85] evaluated the capacity of PCBMA hydrogels to induce three-dimensional mineralization of hydroxyapatite (HA) in comparison with two other zwitterionic equivalents, PSBMA and PMPC. While PCBMA-based hydrogels were able to support HA mineralization, both PSBMA and PMPC produced substantially denser and more extensive mineralization. This was attributed to the lower free water fraction of PCBMA and its reduced ability to maintain a zwitterionic state under the acidic-to-neutral pH changes that occur during the mineralization process, since the carboxyl group is prone to protonation at lower pH. In contrast, the sulfonate and phosphate groups of PSBMA and PMPC preserved their zwitterionic characteristics more effectively, facilitating the sustained recruitment of mineralization precursors and enabling greater mineral incorporation.

PCBMA hydrogels shared excellent cytocompatibility, maintaining the viability of encapsulated stromal cells. Once mineralized, PSBMA and PMPC offered superior retention and controlled release of the osteogenic growth factor rhBMP-2, with \~99% of the protein sequestered after 24 h compared to lower retention in PCBMA composites. Mineralization substantially improved the cell adhesive properties of PSBMA and PMPC, whereas PCBMA-mineralized matrices exhibited comparatively weaker enhancement. Although PCBMA could mediate hydroxyapatite mineralization and provide a cytocompatible scaffold, it was less effective than PSBMA and PMPC in sustaining mineralization under physiological-like conditions.

**Figure 12:**
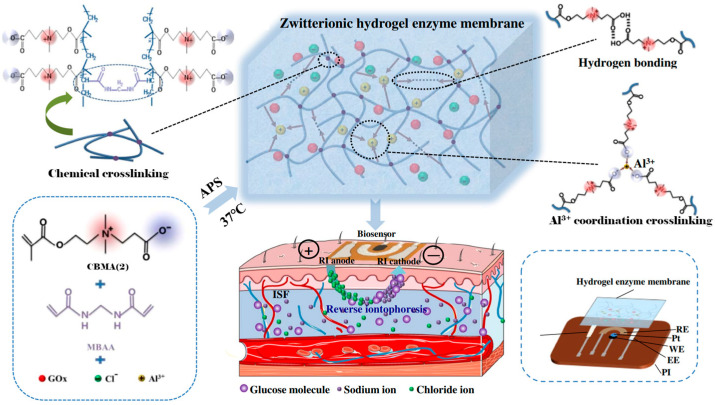
Synthesis and schematic representation of GOx/PCBMA(2)-Al^3+^ hydrogels. Reproduced with permission [86]. Copyright 2024, Springer Nature.

Li et al. [86] developed a glucose biosensor using PCBMA(2) hydrogels (Figure 12). The zwitterionic hydrogel enzyme membrane was created by polymerizing CBMA(2) monomer with MBAA crosslinker and additional crosslinking achieved coordination interactions between Al^3+^ (from AlCl_3_) and -COO^−^. GOx was used as a glucose-recognizing enzyme. The presence of AlCl_3_ introduced a second network through coordination interactions between Al^3+^ and the carboxyl groups of PCBMA(2), increasing the crosslinking density and reducing the pore size of the hydrogel. The increase in GOx/PCBMA(2)-Al^3+^ hydrogel conductivity was 10.4-fold over the plain PCBMA(2) hydrogel, which was attributed to the presence of Al^3+^ and Cl^−^ ions enhancing ion transport within the hydrogel. The hydrogel demonstrated higher catalytic activity for glucose oxidation than free GOx and GOx/PCBMA(2) by preventing enzyme leakage and maintaining the enzyme’s conformation. GOx/PCBMA(2)-Al^3+^ also showed higher sensitivity and a wider linear detection range for glucose compared to the GOx/PCBMA(2) in vitro.

Zeng et al. [87] applied PCBMA hydrogels as electrolytes for high-voltage zinc-ion hybrid capacitors (ZHCs). Synthesis was conducted via free radical photopolymerization of CBMA(2) with an EGDMA crosslinker and an APS radical initiator in deionized water. PSBMA was also synthesized for comparison. PSBMA hydrogels exhibited a much stronger anti-polyelectrolyte effect than PCBMA(2) hydrogels. When immersed in 1 m ZnSO_4_, PSBMA hydrogels swelled from 81% to 246%, whereas PCBMA hydrogels increased only marginally (from 143% to 154%). As a result, PSBMA electrolytes showed an expanded electrochemical stability window (ESW) of 1.88 V, compared to ~1.73 V for PCBMA. Molecular dynamics simulations confirmed that the APE disrupted PSBMA chain self-associations in salt solutions, enhancing chain dispersity, exposing more hydratable ionic groups, and boosting water binding. Incorporation into polyacrylamide hydrogel electrolytes yielded an ESW of 2.58 V and ionic conductivity of 29.3 mS cm^−1^ for PSBMA, compared to 2.10 V and 25.7 mS cm^−1^ for PCBMA. Consequently, zinc-ion hybrid capacitors (ZHCs) with PSBMA electrolytes operated at 2.1 V with a capacity of 188.9 mAh g^−1^ and energy density of 110 Wh kg^−1^, outperforming PCBMA-based ZHCs (1.9 V, 127.4 mAh g^−1^, 82.1 Wh kg^−1^).

Wen et al. [88] combined PCBMA(2) with the REDV peptide to create a hydrogel coating that selectively promotes endothelial cell adhesion. A PCBMA stable hydrogel was formed by photo-crosslinking on titanium (Ti) discs, which is a material commonly used for stents. CBMA(2) monomer was combined with ethylene dimethacrylate crosslinker and 2-hydroxy-2-methylpropiophenone in deionized water. The REDV peptide was then conjugated onto the PCBMA hydrogel via EDC/NHS chemistry. BSA and fibrinogen adsorption was significantly reduced on the REDV/PCBMA surfaces compared to bare Ti surfaces. A substantial decrease for *E. coli* and *S. aureus* bacteria was observed. REDV/PCBMA coatings effectively prevented platelet adhesion and aggregation, prolonged clotting times for the surfaces and led to the hemolysis ratio of the REDV/PCBMA coatings being well below the clinical standard. REDV/PCBMA coatings selectively promoted EC adhesion and proliferation of HUVECs. Higher REDV concentrations led to increased cell density and better cell morphology.

Cabanach et al. [15] fabricated microrobots from zwitterionic hydrogel photoresists, based on CBMA/CBMAX-crosslinker and SBMA/SBMAX-crosslinker. PEG-like microrobots served as control. By varying crosslinker content, swelling, stability, and compressive modulus were tuned, demonstrating that zwitterionic microrobots could achieve sufficient mechanical robustness for manipulation and long-term use. Protein adsorption assays revealed non-detectable fouling on both zwitterionic microrobots, while PEG-like analogs exhibited significant adsorption. In phagocytosis experiments, nearly all PEG-like microrobots were removed by macrophages, whereas PCBMA microrobots showed extremely low uptake (<2%), with one batch reaching ~20% due to synthesis impurities. Extended in-vitro assays demonstrated that PCBMA microrobots could remain undetected by immune cells for up to 90 h.

Chien et al. [89] used a PCBMA hydrogel as a nonfouling platform to investigate how surface-bound RGD conjugation can direct mesenchymal stem cell (MSC) behavior. Low RGD densities preserved MSC stemness by maintaining spheroidal morphology and high expression of pluripotency markers such as Oct4 and Nanog. In contrast, high RGD densities promoted cell spreading, faster proliferation, and robust osteogenic differentiation, as evidenced by elevated alkaline phosphatase activity, bone sialoprotein expression, and calcium deposition. This study highlighted PCBMA’s unique advantage: it not only prevented nonspecific protein adsorption and background cell adhesion but also provided a tunable, highly specific platform for grafting ligands. PCBMA enabled precise dissection of how ligand density influences MSC stemness versus differentiation.

Chien et al. [90] developed a degradable PCBMA hydrogel platform for the selective capture and gentle release of circulating tumor cells (CTCs). PCBMA effectively prevented the adhesion of blood cells and its antifouling nature ensured high specificity when isolating rare CTCs from complex blood samples. The pendant carboxyl groups of PCBMA enabled covalent conjugation of anti-EpCAM antibodies, making the hydrogel able to selectively bind EpCAM-overexpressing tumor cells such as HCT116. The PCBMA hydrogels showed efficient CTC capture, with yields increasing in proportion to antibody density and reaching up to 100% under optimal conditions in cell suspensions. When applied to spiked human blood, the hydrogel retained a capture efficiency of \~45% despite the presence of large numbers of blood cells, underscoring its selective binding capability. Importantly, the degradable crosslinkers in the PCBMA hydrogel allowed for mild, cysteine-triggered dissolution, releasing >94% viable CTCs within 30 min. The released cells retained the ability to adhere and proliferate, demonstrating that the platform preserved cellular integrity.

PCBMA hydrogels have demonstrated excellent antifouling, biocompatibility, and even pro-angiogenic effects in both in vitro and in vivo models. Despite these encouraging results, important limitations remain for PCBMA hydrogels. Most studies have been carried out in small animal models and short implantation periods, leaving their long-term mechanical stability, biodegradation pathways, and performance under physiological stress insufficiently characterized. The scalability of well-controlled hydrogel synthesis also remains challenging and systematic comparisons with clinically used hydrogels such as PEG or PHEMA are still limited in scope. Addressing these gaps will be critical for advancing PCBMA hydrogels toward translational applications in tissue engineering and wound healing.

### 4.4. Membranes for Filtration and Separation

Birkner et al. [91] developed PCBMA-modified polyethersulfone (PES) ultrafiltration membranes by photografting CBMA(2) on PES surfaces. The swelling behavior of PCBMA(2) membranes was significantly influenced by pH due to the protonation of carboxylic acid side groups and showed highly positive zeta potentials at low pH. The water permeability of membranes increased in salt solutions of potassium chloride and potassium perchlorate at pH 3. Ion pair interactions with chaotropic perchlorate ions resulted in pore opening. Reduced dextran fouling was also observed, compared to unmodified membranes. In contrast, PSBMA grafted membranes exhibited neutral or near-neutral zeta potential across the pH range and expanded only as ionic strength increased.

Lin et al. [25] investigated the properties of PCBMA-modified polyvinyl alcohol (PVA) hydrogel membranes. The membranes were produced on a PVA support via photoinitiated electron transfer RAFT polymerization. CBMA(2), CPADB, and Eosin Y photoinitiator and triethylamine in DMF–water solvent were exposed to blue LED light. The PCBMA(2)-modified PVA hydrogel membranes exhibited excellent optical properties, maintaining a transmittance range of 91.2% to 97.0% in the visible spectrum, while thermal stability remained consistent with the unmodified PVA membranes below 150 °C. The swelling ratio of PCBMA(2)-modified PVA hydrogel membranes increased with increasing grafting and the water contact angle decreased compared to unmodified PVA hydrogel membranes. The increased hydrophilicity was also reflected in the increased moisture content. The modified membranes showed non-cytotoxic behavior, with the viability of the eyelid fibroblast cells remaining above 90.55% in all assays and improved anti-protein adsorption capacity compared to the unmodified membranes.

PCBMA-grafted membranes showed strong hydrophilicity, reduced dextran fouling, and high optical transmittance with good in vitro cytocompatibility. However, model fouling tests under static conditions are limited, and the durability of PCBMA-grafted membranes under cross-flow, pressure, and repeated cleaning conditions (e.g., hypochlorite/peroxide) remains unverified. Photografting/ET-RAFT on porous supports may yield non-uniform grafting through pore depth, and scalability to large-area or hollow-fiber formats is not established. The strong pH/salt responsiveness (e.g., pore opening with chaotropic ions at low pH) also raises questions about long-term permeability–selectivity stability in realistic feeds. Biocompatibility data is also limited on cell assays, with no long-term or in vivo membrane safety characterization yet reported.

### 4.5. Grafted Surfaces

PCBMA has been used for grafting to a variety of surfaces for a long time. Zhang et al. [66] prepared PCBMA-grafted surface plasmon resonance (SPR) sensors through a controlled grafting process. Gold-coated glass surfaces were functionalized with self-assembled monolayers (SAMs) and converted into ATRP initiators to be used for grafting PCBMA(2) layers. The reaction took place in a methanol/water (1:1) mixture, with CuBr/bpy as the catalyst and CBMA(2) as the monomer. The thickness of the grafted PCBMA(2) layer ranged between 10 and 15 nm. The PCBMA(2)-grafted surfaces showed very low nonspecific protein adsorption (<0.3 ng/cm^2^) when tested with fibrinogen, lysozyme, and human chorionic gonadotropin (hCG). Immobilization of anti-hCG antibodies on the PCBMA(2) surfaces allowed the surfaces to specifically bind hCG while maintaining resistance to other proteins.

Zhang et al. [92] prepared glass surfaces that were modified with trimethoxysilane and grafted with PCBMA by ATRP polymerization of CBMA(2) monomer in the presence of CuBr/bpy catalysts in a water/methanol mixture (1:1 volume ratio). Fibrinogen adsorption on PCBMA(2)-grafted glass surfaces was reduced to ~4% of that on bare glass (~1.4 ng/cm^2^), comparable to the low levels observed on PEG-like films (4–8%), while untreated glass surfaces adsorbed ~36 ng/cm^2^ fibrinogen. No attachment of bovine aortic endothelial cells was observed on PCBMA(2) surfaces even after 24 h, in contrast to bare glass which supported a nearly confluent cell layer, underscoring the strong antifouling and biomimetic properties of PCBMA coatings.

Zhang et al. [93] also investigated the performance of PCBMA(2)-grafted surfaces under varying ionic strengths and pH conditions. To obtain a Br-terminated ATRP initiator, mercaptoundecyl bromoisobutyrate (Br–thiol SAMs) was grafted on the surface of gold-coated SPR sensor chips or gold-coated silicon chips. PCBMA(2) brushes (10–15 nm) were created on SAMs with the use of CBMA(2) and bpy in a pure water/methanol (1:1 volume ratio) mixture. This provided a good balance of hydrophilicity and antifouling properties to the SAMs, which was superior to other zwitterionic SAMs and PCBAA-grafted SAMs under identical conditions. PCBMA(2)-grafted surfaces exhibited consistently high resistance to fibrinogen adsorption, with levels <0.3 ng/cm^2^ across 3–1000 mM ionic strength, whereas PCBAA- and PEG-grafted surfaces lost antifouling capability under low-salt conditions, showing adsorption >10–20 ng/cm^2^ at 3 mM buffer.

Ladd et al. [94] prepared PCBMA-grafted SAMs on BK-7 glass chips coated with a 2 nm Ti layer and a 48 nm Au layer. Br–thiol SAMs were used as ATRP initiators, along with a CuBr/bpy catalyst polymerization of CBMA(2) monomer in a 1:1 water/methanol mixture. These PCBMA-grafted surfaces exhibited improved resistance to nonspecific protein adsorption compared to PSBMA- and POEGMA-grafted surfaces, with adsorption levels essentially at the detection limit (<3 pg/mm^2^) in both 10% and 100% human serum and plasma, whereas PSBMA and POEGMA surfaces showed adsorption in the hundreds of pg/mm^2^ range and bare glass reached monolayer coverage (~2700 pg/mm^2^).

Cheng et al. [29] developed a switchable polymer surface using CBMA derivatives that integrated antimicrobial and nonfouling properties. The cationic CBMA-EE was polymerized onto gold surfaces through surface-initiated atom transfer radical polymerization (SI-ATRP). Gold-coated substrates with SAMs were immersed in DMF containing CBMA-EE, CuBr, and HMTETA, which resulted in 26 to 32 nm coatings. Hydrolysis of PCBMA-EE converted the cationic into a zwitterionic PCBMA(1) surface, enabling a reversible switch between antibacterial activity and nonfouling functionality. The hydrolysis rate, influenced by pH and temperature, was optimized to physiological conditions (37 °C, pH 10.0) for practical applications.

The same methodology was applied for PCBMA(2) grafting on gold surfaces. PCBMA(2) grafts exhibited ultralow fouling behavior and negligible bacterial attachment, which was even less in the case of cationic PCBMA-EE or permanently cationic poly(methacryloyloxyethyldimethyloctylammonium bromide) (PC8NMA), which was used as a control to represent conventional antimicrobial surfaces. PCBMA(2) was also non-switchable and remained in its zwitterionic form.

Zhang et al. [95] evaluated blood compatibility of surfaces coated with PCBMA brushes. CBMA(2) was grafted on gold surfaces using ATRP initiated from bromoisobutyrate-functionalized SAMs. The thickness of the PCBMA(2) brushes was 10–15 nm. The brushes exhibited the lowest protein adsorption from undiluted human plasma, with PCBMA surfaces adsorbing only 0.4 ± 0.9 ng/cm^2^, compared to 9.1 ± 0.6 ng/cm^2^ for PSBMA and 9.2 ± 6.5 ng/cm^2^ for POEGMA brushes. In contrast, all initial SAMs exhibited significantly higher adsorption (>250–300 ng/cm^2^), even though they resisted fibrinogen from buffer. PCBMA, PSBMA, and POEGMA brushes all significantly reduced platelet adhesion compared to uncoated polystyrene (~148–211 × 10^3^ platelets/cm^2^), with PCBMA brushes supporting only 4.7 × 10^3^ platelets/cm^2^, similar to PSBMA (5.8 × 10^3^) and lower than POEGMA (9.6 × 10^3^). Among the tested surfaces, PCBMA showed comparable or better performance than the widely studied PSBMA and POEGMA brushes, combining ultralow protein adsorption with minimal platelet adhesion.

A study of blood plasma interactions with PCBMA-coated gold surfaces was conducted also by Emmenegger et al. [96]. CBMA(2) was polymerized via ATRP on Br–thiol SAMs with the use of CuBr and Bpy in a water/methanol (1:1) mixture. PCBMA completely prevented plasma deposition on the surface, with SPR showing no detectable fouling (<6 pg/mm^2^), while PSBMA and PCMA coatings, though effective against single-protein adsorption (HSA, IgG, fibrinogen, and lysozyme ~0 ng/cm^2^), still accumulated substantial plasma deposits (~16 and ~23 Δλres nm, respectively).

Cheng et al. [97] prepared PCBMA surfaces grafted on glass via ATRP of CBMA(2) under the use of CuBr, CuBr_2_, and bpy as the catalyst system for the development of antifouling and antibacterial films. PCBMA surfaces exhibited minimal nonspecific protein adsorption compared to unmodified glass and tissue culture polystyrene substrates. PCBMA also significantly reduced biofilm formation of *Pseudomonas aeruginosa* PAO1 and *Pseudomonas putida* on the surfaces.

Brault et al. [98] used PCBMA for creating ultralow fouling and functionalizable coatings on SiO_2_ surfaces of SPR chips by grafting their surfaces with PCBMA(2) chains equipped with the initial group of 3,4-dihydroxy-L-phenylalanine (DOPA). Carboxylic acid groups in PCBMA(2) were activated by NHS and EDC to form an NHS ester intermediate for reaction with the primary amine groups present on antibodies. The zwitterionic PCBMA layer effectively resisted nonspecific protein adsorption in human plasma and serum. The functionalization of PCBMA surfaces with anti-activated leukocyte cell adhesion molecule (ALCAM) antibodies enabled specific detection of the ALCAM cancer biomarker directly from undiluted human serum with high sensitivity.

**Figure 13:**
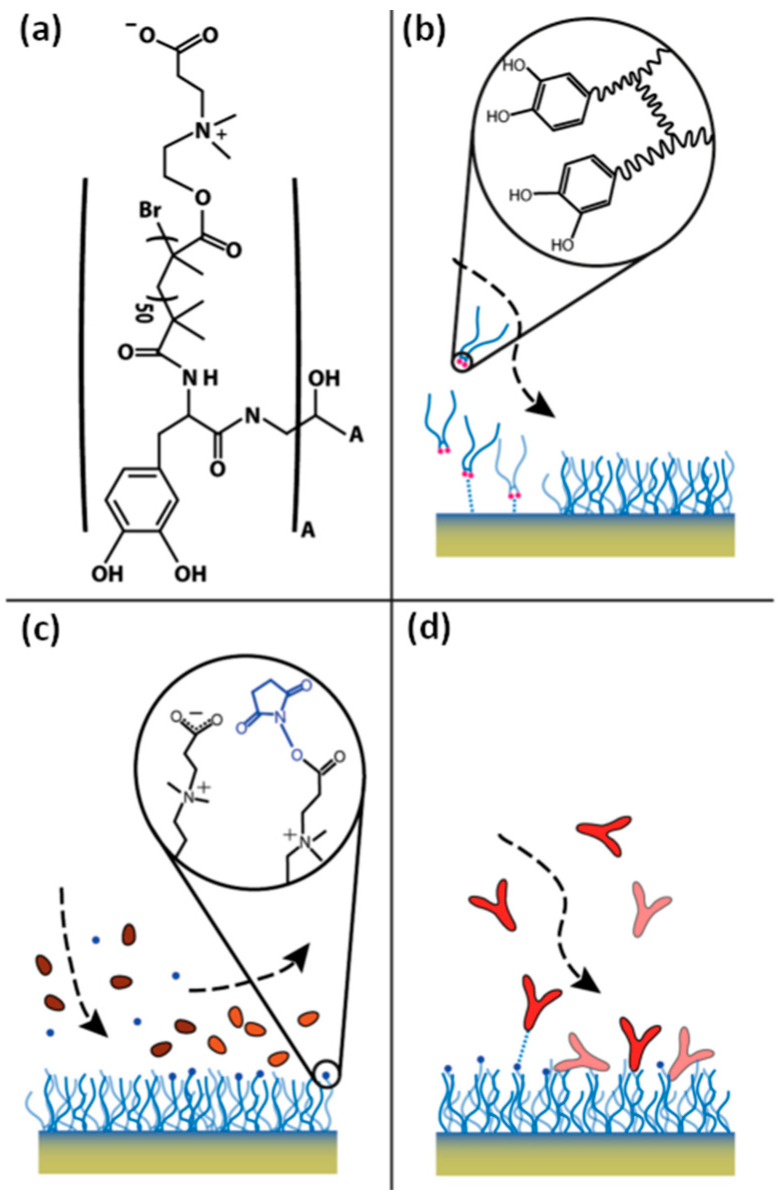
(**a**) Structure of DOPA-PCBMA(2), (**b**) direct adsorption onto the SiO layer of the microcantilever, (**c**) conversion of carboxyl groups to NHS esters, and (**d**) attachment of IgG antibodies on the NHS esters. Reproduced with permission [99]. Copyright 2010, American Chemical Society.

Von Muhlen et al. [99] created a DOPA-PCBMA(2) coating to enhance the performance of suspended microchannel resonators (SMRs) in biomarker detection. The PCBMA(2) layer was formed by attaching ATRP-synthesized PCBMA conjugated with DOPA onto the SiO_2_ microcantilever surface. Terminal carboxylic groups of PCBMA were used to covalently bind the immunoglobulin G (IgG) antibodies by the NHS/EDC chemistry utilizing the primary amine groups of the antibodies. PCBMA(2)-coated SMRs successfully detected ALCAM, a cancer biomarker, at physiologically relevant concentrations (10–1000 ng/mL) in undiluted mammalian serum. The results showed a dose–response relationship with high specificity, which was enabled by the zwitterionic coating’s resistance to nonspecific adsorption. The procedures are shown in Figure 13.

Krause et al. [100] prepared PCBMA films on gold substrates using surface-initiated photoiniferter-mediated polymerization (SI-PIMP). SAMs of dithiocarbamate photoiniferter were exposed to UV light in the presence of the CBMA(2) or CBAA monomers under nitrogen. Both PCBMA and PCBAA films exhibited ultralow fouling properties with nonspecific protein adsorption from undiluted human plasma. Films were functionalized using EDC/NHS chemistry enabling antibody (anti-TSH) immobilization. Despite differences in film thickness, both PCBMA and PCBAA coatings demonstrated excellent post-functionalization nonfouling properties, with protein adsorption remaining as low as ~4.2 ng/cm^2^ before and ~8.8 ng/cm^2^ after antibody immobilization, while supporting specific antigen detection from undiluted human plasma at concentrations down to 1 ng/mL.

Mahmud et al. [101] focused on the synthesis and application of PCBMA films as bioresistant surfaces. PCBMA(2) was obtained by ATRP in a water/methanol mixture and grafted onto micro-etched gold substrates. The PCBMA(2) films demonstrated strong resistance to cell adhesion and protein adsorption was comparable to that of the well-established SAMs of oligo(ethylene glycol) alkanethiolates (EG6). However, contrary to EG6 they showed excellent stability, maintaining their properties even after two weeks of storage. PCBMA(2)-grafted surfaces provided nearly 90% cell adhesion resistance for various cell types for up to 14 days. Compared to EG6 SAMs, PCBMA films were highlighted as more cost-effective and easier to handle, making them a practical choice for research and industrial applications.

Hu et al. [102] prepared antimicrobial and antiadhesive surfaces using PCBMA(2) grafted on gold-coated glass substrates via SI-ATRP. The polymer layer had a thickness of ~59.5 nm. Well-dispersed silver nanoparticles were formed by soaking the polymer in silver nitrate solution and in situ reduction. The surface killed over 99.8% of Escherichia coli K12 within one hour and 98.7% of dead bacteria was released after gentle washing in PBS.

Kirk et al. [103] demonstrated the use of pre-grown PCBMA(2)-DOPA grafted onto silicon microring resonators to enable label-free biosensing in undiluted human plasma. The PCBMA(2)-DOPA coatings effectively reduced protein adsorption from undiluted human plasma retained even after monoclonal anti-streptavidin antibody immobilization. Streptavidin detection was demonstrated in both buffer and plasma with the sensors achieving enzyme-linked immunosorbent assay sensitivity.

Yang et al. [104] prepared PCBMA-coated gold nanoparticles (PCBMA-GNPs) as an alternative to conventional PEG-coated nanoparticles (PEG-GNPs) for drug delivery applications. The PCBMA(1)-GNPs were synthesized by SI-ATRP grafting of tBuCBMA monomer onto 11-mercaptoundecyl 2-bromoisobutyrate-functionalized GNPs followed by acid hydrolysis to remove the tert-butyl protecting groups. PCBMA(1)-GNPs and PEG-GNPs had nearly identical sizes (~40 nm) and neutral zeta potentials at pH 7.4. Unlike PEG-GNPs, which aggregated in undiluted human plasma (~300 nm in diameter), PCBMA-GNPs resisted protein adsorption and remained stable. In vivo, PCBMA-GNPs showed prolonged circulation, with half-lives of 55.8 h after the first injection and 55.6 h after the second, compared to 8.7 h and 5.2 h for PEG-GNPs, which exhibited the accelerated blood clearance (ABC) phenomenon. Antibody assays confirmed that PEG-GNPs triggered strong immune responses (IgM increased~14-fold; IgG increased ~3.8-fold over baseline), whereas PCBMA-GNPs produced no detectable increase in IgM or IgG. Biodistribution studies further showed that PEG-GNPs accumulated in liver and spleen, while PCBMA-GNPs remained in circulation for days, highlighting the superior stealth and immune-evasive properties of PCBMA coatings.

Zhu et al. [105] modified cellulose paper with PCBMA by SI-ATRP of CBMA(2). The hydroxyl groups on the cellulose surface were initially converted into ATRP initiators and then reacted with CBMA(2) in the presence of CuBr and bpy ligand in a 1:1 methanol/water mixture. PCBMA-modified cellulose paper used for glucose detection displayed faster diffusion of liquids through the hydrophilic channels, improved transport of analytes to the designated detection zones, and demonstrated greater sensitivity in both buffer and undiluted human serum compared to unmodified cellulose. Anti-BSA and anti-fibrinogen antibodies were covalently immobilized on PCBMA-modified paper. The functionalized paper specifically detected the corresponding fluorescence-labelled antigens (BSA and fibrinogen), while resisting nonspecific binding of other molecules.

Wang et al. [106] developed nylon surfaces modified with CBMA(2) monolayers for capturing circulating tumor cells (CTCs) in vivo. 3-Aminopropyltriethoxysilane (γ-APS) was utilized as a coupling reagent to graft PCBMA(2) monolayers onto the nylon surface via Michael addition. The PCBMA(2)-modified nylon surface showed enhanced wettability, a lower amount of absorbed BSA, and better anticoagulation properties than pristine nylon. No negative impact on cell viability or morphology was observed, confirming that the PCBMA-grafted nylon is safe for in vivo applications. The functionalized nylon surface had a much higher affinity for EpCAM-positive tumor cells compared to pristine nylon, effectively capturing a larger number of target cells. Nylon wire modified by the immobilization of anti-EpCAM antibodies using EDC/NHS chemistry showed extreme efficacy and functionality by successfully capturing CTCs, in comparison to the unmodified wire that did not capture any of them.

Chen et al. [107] designed silica nanoparticles incorporating PCBMA brushes for tuning protein adsorption. The synthesis was carried out by polymerizing DMAEMA using various RAFT CTAs immobilized on the surface of silica nanoparticles. The end groups of the PDMAEMA chains were quaternized by reacting them with β-propiolactone. This reaction converted the amine groups to quaternary ammonium groups, resulting in the formation of P(DMAEMA-co-CBMA(2)). The degree of quaternization varied between 60% and 100%, with higher quaternization levels leading to a marked reduction in protein adsorption. Acidic and basic protein adsorption ability depended on the quaternization degree of grafted chains.

Zhang et al. [108] synthesized PCBMA-grafted silica nanoparticles for enzyme immobilization. Silica nanoparticles were functionalized with amino groups using (3-aminopropyl)triethoxysilane, followed by the introduction of BIBB ATRP initiator for polymerization of CBMA(2). Silica nanoparticles (SNPs)-PGMA were prepared for comparison. Catalase and lipase complexed on SNPs-PCBMA exhibited higher thermal and storage stability than those on SNPs-PGMA. Following incubation at 50 °C for 20 min, PCBMA-CAT and PCBMA-CRL retained 60.5% and 60.3% activity, respectively, compared to 55.6% and 48.3% for PGMA conjugates. Storage stability was also improved, with PCBMA-immobilized enzymes maintaining ~50–80% activity after 2–7 weeks at 4 °C, whereas free enzymes rapidly lost activity (>90% loss for catalase in 15 days). Both immobilized systems showed good reusability, retaining ~50–60% activity after eight cycles, but PCBMA conferred greater protection under neutral and alkaline conditions. Despite slightly lower protein loading (15.0 mg/g for catalase and 2.7 mg/g for lipase vs. 26.4 and 7.4 mg/g for PGMA), the superhydrophilic PCBMA shell preserved enzyme conformation and stability more effectively.

Wang et al. [109] combined the high conductivity of polyaniline (PANI) with the antifouling properties of PCBMA. Glassy carbon electrodes were initially functionalized with PANI nanowires via electrodeposition. Then, the nanowires were incubated in a solution containing CBMA(2), EDC, and NHS to covalently attach CBMA(2) monomers to the PANI surface. The thicknesses of the PCBMA layer increased from 50 to 150 nm as a result of photopolymerization. PCBMA/PANI nanowires exhibited significantly improved hydrophilicity compared to unmodified PANI nanowires, with a low contact angle of 12.5°, indicating good wettability.

Ukita et al. [110] studied the efficacy of PCBMA coatings in reducing thrombosis on artificial lung surfaces. Three different methods of applying PCBMA and PCBAA copolymer coatings to artificial lung surfaces were reported: (1) A “grafting-to” approach resulting in DOPA-PCBMA-coated surfaces was investigated. In this synthesis, DOPA-PCBMA conjugates were created by polymerizing CBMA(1) using a DOPA-containing initiator, HMTETA, and CuBr in methanol. The surface coating was carried out in a water/methanol solution (80%/20% *v*/*v*) containing TRIS salt. (2) A “grafting-from” approach by Activators ReGenerated by Electron Transfer (ARGET) ATRP was investigated, using CuBr_2_ and bpy in a water/methanol mixture to prepare PCBMA coatings on the artificial lung surfaces. (3) A “grafting-to” approach using PCBAA copolymerized with hydrophobic monomers for adhesion served as a comparison, alongside ARGET-ATRP coatings. In vivo sheep studies (36 h, no continuous anticoagulation) showed that DOPA-PCBMA-coated devices had the lowest blood flow resistance and failure rate (25% vs. 40% for ARGET-ATRP, 60% for uncoated, and 80% for copolymer-coated devices). In a 4 h rabbit model at clinically relevant ACTs, DOPA-PCBMA coatings reduced fibrin formation (*p* = 0.06) and achieved a 59% reduction in thrombus weight compared to uncoated devices (*p* < 0.05). SEM confirmed suppression of clot deposition across both hollow fibers and weaving fibers. These results demonstrate that DOPA-PCBMA coatings provided improved antithrombotic performance compared to hydrophobic copolymers and ARGET-ATRP coatings.

Qiao et al. [111] prepared PCBMA-functionalized gold nanorods (PCBMA-AuNRs), which exhibited pH-induced surface charge-switchable activities to enhance their antibacterial efficacy. Gold nanorods were coated with hexadecyl trimethyl ammonium bromide and RAFT polymerization of t-BuCBMA was carried out using AIBN in DMF. The removal of t-Bu groups was carried out with TFA. The PCBMA-AuNRs demonstrated a transition from a negative charge at physiological pH (around 7.4) to a positive charge under acidic conditions (pH around 5.5), which is conducive to bacterial infections. PCBMA-AuNRs exhibited significantly higher antibacterial activity against both *E. coli* and *S. aureus* compared to PEGMA-coated AuNRs, achieving >97% killing within 3 min of NIR irradiation at 808 nm, while PEGMA-AuNRs left substantial surviving colonies. Against drug-resistant strains, PCBMA-AuNRs killed >90% of MRSA and ESBL *E. coli* after 5 min of irradiation, outperforming PEGMA-coated controls. In mature *S. aureus* biofilms, PCBMA-AuNRs reduced viability to <10% after 8 h of treatment plus 5 min irradiation, compared to ~40% survival with PEGMA-AuNRs. Confocal microscopy confirmed deep penetration of PCBMA-AuNRs through the biofilm matrix, enabling uniform killing across the full thickness, whereas PEGMA-AuNRs remained largely at the surface.

Naito et al. [112] presented a novel approach to reduce clot formation in artificial organs using PCBMA coating in combination with systemic administration of a selective factor XII inhibitor (FXII900). The study was designed to avoid the use of heparin which, while reducing the risk of clot formation, can nevertheless cause bleeding and other undesirable effects. Initially DOPA ATRP-initiator was used, along with CuBr and HMTETA for the polymerization of CBMA(2) in methanol. The resulting DOPA-PCBMA adduct formed the surface of polysiloxane-coated polypropylene fibers, which created the main part of a model of an artificial, extracorporeal organ in contact with circulating blood. Surfaces coated with DOPA-PCBMA adduct with systemic administration of a new anticoagulation peptide (FXII900) instead of heparin significantly reduced clot formation and decreased blood flow resistance compared to both heparin alone and DOPA-PCBMA with the presence of heparin. They also provided a normal bleeding time, in contrast to the prolonged bleeding time observed for DOPA-PCBMA surfaces and used with heparin.

Christau et al. [113] developed macrophage-targeting PLGA nanoparticles coated with PCBMA to enhance their stability and targeting capabilities. PLGA nanoparticles were synthesized using different PLGA/poly(l-lactide) (PLA) ratios and coated with PCBMA via Si-ATRP. The PCBMA-coated nanoparticles were further functionalized with NHS-modified mannose using EDC. PCBMA–mannose-coated PLGA nanoparticles exhibited the smallest change in zeta potential upon incubation in HSA (Δ = 9.3 mV, from −33.6 to −14.2 mV), compared to PEG-coated (Δ = 21.0 mV) and mannose monolayer-coated nanoparticles (Δ = 13.5 mV), suggesting reduced protein adsorption and improved colloidal stability. In plasma, their hydrodynamic size was ~130 nm smaller than PEGylated particles at early time points, which is consistent with a smaller protein corona, and nanoparticle tracking analysis showed comparable aggregation levels to PEG (PCBMA–mannose: 762 ± 172 aggregates per 10^5^ particles, PEG: 763 ± 140), while mannose monolayer particles aggregated more strongly (939 ± 250). PCBMA–mannose nanoparticles displayed ~4-fold higher uptake by RAW 264.7 macrophages than PEG- or mannose monolayer-coated NPs, owing to the high density of mannose groups presented in the PCBMA brush shell and more effective engagement of macrophage mannose receptors.

PCBMA-based coatings demonstrated excellent blood compatibility, with ultralow protein adsorption, reduced platelet adhesion, and suppressed thrombosis compared to PEG, PSBMA, and PCMA surfaces. However, many of these results derive from short-term or small animal studies and their durability under chronic implantation, repeated blood exposure, and mechanical stress has not been fully established. Surface-initiated ATRP or RAFT processes used for grafting are difficult to scale and long-term regulatory data on degradation products and safety are lacking. Switchable or multifunctional PCBMA coatings, while conceptually powerful, add complexity that may hinder reproducibility in clinical devices. Addressing these challenges will be essential to advance PCBMA-based blood-contacting materials from laboratory studies to clinical translation.

## 5. Molecular Dynamics of Poly(Carboxybetaine Methacrylate)

Simulations and molecular dynamics (MD) studies have focused on investigating the molecular origins of hydration and antifouling properties of PCBMA. Most MD studies on PCBMA employed all-atom explicit solvent models, typically using standard simulation packages to capture hydration and conformational behavior with high fidelity. Polymer chains of about 10–20 CBMA units were often modeled, corresponding to molar masses of a few kilodaltons, while hydrogel models were built from crosslinked PCBMA chains of similar lengths. These systems were solvated with thousands of explicit water molecules, and simulated for tens to hundreds of nanoseconds, sufficient to probe hydration shell formation, conformational stability, zwitterion–water interactions, and in some cases zwitterion–zwitterion associations. Quantum chemical approaches were used to refine force-field charges and analyze hydration energetics. The simulated conditions were chosen to mimic aqueous environments relevant to biomedical use, thereby providing insights into hydration-dependent transitions, conformational dynamics, and antifouling properties of PCBMA.

He et al. [114] employed MD simulations to investigate the hydration-dependent behavior of PCBMA(2) hydrogels at various swelling states. A detailed all-atom model of the hydrogel was constructed, incorporating three main polymer chains of 15 CBMA(2) repeating units each, interconnected by 1,3-dioxane cyclic crosslinkers. Additional shorter chains, each containing five CBMA(2) units, linked the main chains to form a three-dimensional network. Hydrogels were simulated with varying water contents, ranging from 28% to 91% by weight, to understand hydration effects on the polymer network and zwitterionic interactions.

Three distinct transitions appeared as the water content increased. The first between 33% and 37% water content, expressed through a change in the self-diffusion coefficient of water, indicated the saturation of hydration shells around the zwitterionic side chains of CBMA. Any additional water over and above this limit became less restricted and was able to move more freely. The second transition, at 62% water content, represented the equilibrium swelling state of the hydrogel. At this point, the zwitterionic physical crosslinks formed by inter- or intrachain associations of zwitterionic side groups become weaker and less stable. As the water content increases, these zwitterionic interactions break down, reducing the number of crosslinks that hold the hydrogel structure together. The third transition was identified at 81% water content, where the polymer network reached its upper limit swelling state. Significant increases in bond and angle interaction energies within the polymer network reflect strain on the chemical bonds due to maximum chain extension.

He et al. [115] investigated the mechanical properties of PCBMA hydrogels using MD simulations. Two hydrogel systems were compared: conventional PCBMA(2) hydrogels and hydroxyl-modified PCBMA (OH-PCBMA) hydrogels. These hydrogels contained hydroxyl groups between the quaternary ammonium and carboxylate groups of the zwitterionic repeating unit. These hydroxyl groups acted as additional physical crosslinkers, forming hydrogen bonds with carboxylate groups within the polymer network. The simulations revealed that OH-PCBMA hydrogels exhibited a higher elastic modulus compared to PCBMA(2) hydrogels at their respective equilibrium water contents. This enhancement was attributed to the formation of hydrogen bonds in OH-PCBMA, which strengthened the polymer network. Additionally, the equilibrium water content of OH-PCBMA hydrogels was lower than that of PCBMA(2) hydrogels, contributing to their improved mechanical performance. Further analysis also showed that OH-PCBMA hydrogels had a smaller root-mean-square deviation (rmsd) of zwitterionic side-chain heavy atoms, indicating a more stable and tightly connected network.

Shao and Jiang [116] estimated the effect of carbon spacer length on the properties of zwitterionic carboxybetaines in general, using MD and quantum chemical calculations. Carboxybetaines molecules, composed of a cationic trimethyl ammonium group and an anionic carboxylate group linked by methylene units, were studied with spacer lengths ranging from zero to four carbons. Shorter spacer length leads to strong electrostatic interactions between the charged groups, resulting in reduced hydration and ionic association. As the length increases, the interplay between the charged groups diminishes, leading to enhanced hydration and ionic association stability. When spacer length is three or more, the charged groups exhibited consistent hydration properties and partial charges, suggesting that the effect of length becomes negligible.

Shao et al. [117] compared how charge differences of two zwitterionic polymers PSBMA and PCBMA influence their interaction and respond to changes in temperature and salt levels. PSBMA formed stronger connections between its polymer chains, shown by higher storage (G′) and loss (G″) moduli. These connections weakened as the temperature increased. In contrast, PCBMA had fewer connections, and its behavior remained stable even when the temperature changed. The hydrodynamic radius of PSBMA and PCBMA were measured in water in different NaCl concentrations. The R_h_ of PSBMA decreases with increasing salt concentration to a limiting value and then remains stable, indicating that a balance between association and dissociation of zwitterionic groups has been achieved. In contrast, PCBMA polymers show minimal changes in R_h_ at different salt concentrations, suggesting weaker association interactions and greater structural stability in saline environments.

Molecular dynamic simulations confirmed these findings by looking at how the positive and negative parts of the polymers interacted. MD showed that PCBMA polymers had fewer and less persistent N-O pairs compared to PSBMA polymers, resulting in weaker associations in PCBMA. The differences in these associations were attributed to the charge density of the cationic and anionic groups. PCBMA polymers, having different charge densities of the groups, showed weaker associations, while PSBMA polymers, with similar charge densities in their cationic and anionic groups, demonstrated stronger associations.

Conformational behavior and hydration dynamics of PCBMA were explored by Zhu et al. [118] using MD simulations and compared with PEG to provide deeper insights into the unique antifouling properties of PCBMA. Models of PCBMA containing 10 CBMA units and PEG with fifty-five ethylene glycol units were constructed in the simulations to achieve comparable molar masses. PEG exhibited a flexible, dynamic, and curled conformation with substantial segment-to-segment interactions, leading to a hindered hydration effect (SASA 34.5 ± 3.3 nm^2^). In contrast, PCBMA displayed hierarchical flexibility with a fixed backbone and relatively free sidechains, allowing each zwitterionic sidechain to interact independently with water molecules (SASA 30.2 ± 1.1 nm^2^). This structural organization created an exclusive hydration volume for each sidechain and more extended water interactions. PCBMA retained water effectively, with hydration residence times of ~31.8 ps compared to 17.3 ps for PEG, and hydrogen bond lifetimes of 29.8 ps versus 21.5 ps. Radial distribution analysis distinguished three hydration layers around PCBMA, with the innermost layer reaching 3.25× the bulk water density, while PEG displayed only two layers. Umbrella sampling confirmed that PCBMA had a more negative hydration free energy (−100 kcal/mol) than PEG (−63.7 kcal/mol), indicating stronger water affinity and explaining its superior antifouling performance.

**Figure 14:**
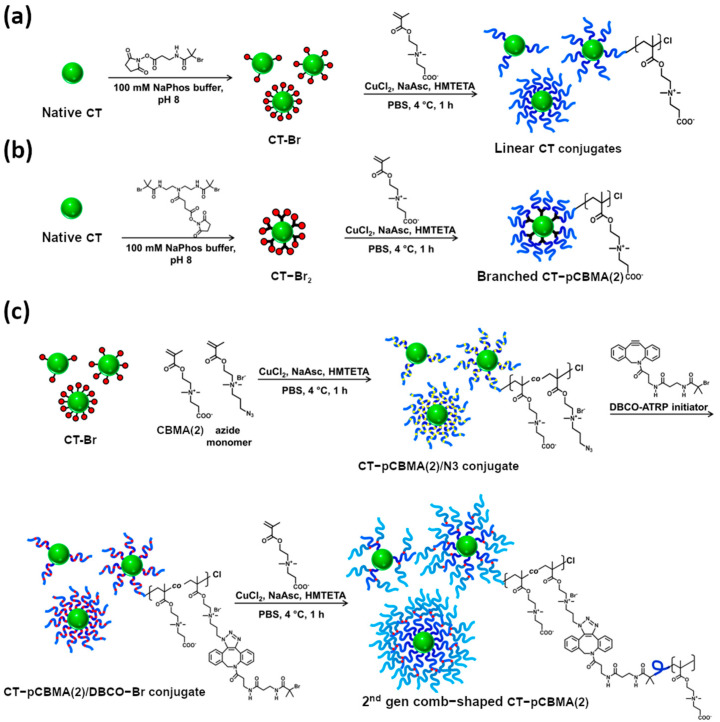
Synthetic routes of (**a**) linear, (**b**) branched, and (**c**) comb-shaped CT-PCBMA(2) conjugates via ATRP. Reproduced with permission [119]. Copyright 2021, American Chemical Society.

Kaupbayeva et al. [119] presented comprehensive experimental and MD studies on the synthesis and characterization of chymotrypsin PCBMA(2) conjugates (CT-PCBMA(2)). A series of CT-PCBMA(2) conjugates of linear, branched, and comb-shaped configurations were prepared as shown in Figure 14. ATRP initiators were attached to specific sites on the chymotrypsin surface, followed by the polymerization of PCBMA(2) chains using CuCl_2_, NaAsc, and HMTETA in PBS.

The comb-shaped polymers were designed with long backbones and side chains to form dense molecular sieves around the enzyme. They exhibited a spherical shape with high molar masses and higher hydrodynamic radii compared to linear and branched conjugates. The comb-shaped conjugates contained nearly 99% polymer by mass, forming a dense nanoarmor around the enzyme. The catalytic efficiency of the conjugates was reduced compared to the native enzyme but they retained substantial activity, indicating that the substrate was partially accessible to the active site.

Molecular dynamics predicted that the dense structure formed by the comb-shaped polymers would effectively shield the enzyme from protein inhibitors while allowing small molecule substrates to access the active site. The CT-PCBMA(2) conjugates exhibited remarkable resistance to inhibition by protein inhibitors and maintained substantial enzymatic activity, even in the presence of up to a 100-fold molar excess of inhibitors.

Molecular dynamics simulations have provided valuable mechanistic insights into the hydration, conformational stability, and antifouling properties of PCBMA, offering explanations for its improved performance compared to PEG and other zwitterionic polymers. These studies clarified how hydration layers, spacer lengths, and charge distributions influence protein resistance and structural stability, and how PCBMA conjugates can shield enzymes or improve hydrogel mechanics. Despite these results, MD investigations remain limited to relatively short timescales, small system sizes, and simplified models that may not fully capture physiological complexity. Long-term degradation, interactions with diverse plasma proteins, and multiscale processes under flow remain underexplored. Bridging these atomistic simulations with experimental validation and coarse-grained or continuum models will be necessary to translate computational insights into predictive design rules for biomedical applications.

## 6. Conclusions and Future Outlook

The synthesis of PCBMA has shown significant advancements during the last 20 years. Polymerization techniques, such as free radical, RAFT, and ATRP were employed to synthesize PCBMA and its derivatives, offering control over the polymer’s architecture and functionality. Free radical polymerization was mostly adapted for synthesizing PCBMA nanogels and hydrogels, while ATRP and RAFT polymerization were utilized for synthesizing well-defined block copolymers, star copolymers, and grafted structures. Surface-initiated ATRP or RAFT polymerization was used for grafting PCBMA onto various substrates, such as glass, silica, or gold nanoparticles. Click chemistry was also utilized as a post-polymerization modification technique that enhanced the functionality of PCBMA-based materials by enabling the efficient attachment of various functional groups.

PCBMA has emerged as a promising alternative to PEG, due to its strong hydration and antifouling properties, biocompatibility, and responsiveness to environmental stimuli. Through different architectures, such as homopolymers, block and star copolymers, hydrogels, nanogels, and surface coatings, PCBMA-based materials have been utilized in drug delivery, regenerative medicine, and antifouling surface design.

**Hydration and Antifouling Behavior:** PCBMA demonstrated strong antifouling performance, which is attributed to its dense hydration layer and balanced zwitterionic charge across all architectures presented in this review. Molecular dynamics (MD) simulations confirmed stable and dynamically structured hydration, which explains PCBMA’s lower protein adsorption relative to PSBMA or PMPC analogs.

**Drug/Gene Delivery:** PCBMA-based nanocarriers were utilized for gene and drug delivery applications. The zwitterionic shell offered excellent colloidal stability, prolonged circulation time, and reduced immunogenicity compared to PEG or PEI analogs. PCBMA’s strong hydration layer provided a stealth-like effect that minimized opsonization and systemic clearance by the mononuclear phagocyte system, thus allowing more payload to reach target tissues. By incorporating pH-sensitive/degradable hydrophobic blocks, researchers managed to achieve endosomal escape and controlled release of the encapsulated drug within the cytosol. In gene delivery systems, PCBMA-containing vectors demonstrated efficient complexation and protection of nucleic acids, while promoting transfection with significantly lower cytotoxicity compared to PEI-based systems. These properties demonstrated the dual functionality of PCBMA, enhancing systemic circulation and protection of nucleic acids while also enabling effective intracellular delivery.

**Bioconjugates and Protein Therapeutics:** PCBMA enhanced the pharmacological performance of proteins and enzymes in bioconjugate polymers. Conjugation with PCBMA effectively reduced nonspecific protein interactions and protected biomacromolecules from rapid clearance, leading to prolonged circulation times. PCBMA also preserved the structure and catalytic activity of the conjugated proteins.

**Regenerative and Tissue Engineering Platforms:** Hydrogels incorporating PCBMA—either as homopolymer networks or copolymer blends—have been used in regenerative medicine, tissue scaffolding, and wound healing applications. Their zwitterionic character enabled them to form highly hydrated, nonfouling matrices that reduce unwanted protein adsorption and cellular adhesion, and absorb wound exudate while supporting an anti-inflammatory environment for tissue compatibility. PCBMA hydrogels exhibited excellent cytocompatibility and low immunogenicity. Hydrogels responsive to pH, redox, or enzymatic triggers, led to smart release of bioactive compounds in situ. Careful incorporation of selected crosslinkers or other comonomers tuned the mechanical properties of the PCBMA hydrogels, creating either soft, injectable gels or more robust scaffolds.

**Surfaces and Coatings:** PCBMA-based coatings exhibited ultralow protein adsorption and resistance to cellular adhesion. Hydration layer played a key role in suppressing nonspecific interactions with biological molecules, maintaining this ability across a range of grafting densities and chain architectures. PCBMA-based coatings have also been applied to biosensor surfaces, where their antifouling properties improve signal clarity by minimizing background noise. Architectures such as bottlebrush grafts and block copolymer interfaces could be modified in thickness and hydration density.

**Molecular Design Principles and Simulation Insights:** MD simulations further investigated and explained PCBMA’s solvation behavior, conformational dynamics, and zwitterion–water interactions. Integrating simulation with experimental rheology, SAXS/DLS, or cell uptake studies will strengthen future material design.

PCBMA demonstrated strong antifouling performance and promising performance in biomedical applications, but several challenges remain before clinical translation. While controlled radical methods, such as RAFT and ATRP, enable precise architectures, they are costly and sensitive to reaction conditions, leading to complicated reproducibility and limited scalability for large-scale manufacturing. Purification and removal of catalysts/ligands is also essential to meet pharmaceutical standards, adding to production complexity. From a regulatory standpoint, PCBMA is not yet established as a safe excipient and long-term studies on immunogenicity, biodegradation, and systemic clearance are required before approval by major regulatory agencies. Addressing these challenges will require not only optimized synthetic protocols, but also systematic preclinical evaluations. Long-term safety studies are still required for PCBMA and its environmental behavior also deserves attention. Unlike PEG, whose persistence in wastewater has been investigated, little is known about the biodegradability or accumulation of zwitterionic polymers such as PCBMA in natural ecosystems. Addressing these regulatory and environmental uncertainties will be crucial for advancing PCBMA materials toward sustainable and clinically acceptable applications.

PCBMA represents one of the most promising alternatives to PEG, uniquely combining ultralow fouling with excellent biocompatibility and functional versatility. While important challenges remain in synthesis, scalability, regulation, and environmental assessment, the growing body of evidence suggests that PCBMA-based materials have the potential to significantly impact future biomedical applications. With continued progress, PCBMA is well-positioned to become a next-generation platform polymer for antifouling and therapeutic systems.

## Figures and Tables

**Table 1:** Monomer synthesis and properties.

Monomer	Yield	Synthesis Notes/Purification	Solubility Profile	Stability/pH Responsiveness	Ref.
**CBMA(2)**	88% [24]; ~75% [25]	Prepared by reaction of DMAEMA with β-propiolactone (Liaw et al.) [24]; acrylic acid and 4-methoxyphenol in acetone [25]; purified by precipitation/solvent washing	Water, alcohols, DMSO; slightly soluble in DMF and dimethylacetamide	Stable under neutral conditions; hydrolyzes under strong acidic/basic environments (as a betaine structure)	Liaw et al. [24], Lin et al. [25]
**t-BuCBMA**	96%	Reaction of DMAEMA with tert-butyl bromoacetate in acetonitrile; product precipitated with ether and dried	Acetonitrile, DMF, and water	Stable in organic solvents; tert-butyl ester groups quantitatively hydrolyzed by TFA within 1 h to yield CBMA(1)	Cao et al. [26]
**CBMA(1)**	-	Can be obtained by hydrolysis of t-BuCBMA or CBMA-EE using TFA, followed by ion-exchange resin purification	Water, slightly soluble in acetonitrile	Zwitterionic form with C1 spacer; stable under neutral aqueous conditions	Mai et al. [27]
**CBMA-OH**	96%	Prepared from sarcosine tert-butyl ester + glycidyl methacrylate and then TFA deprotection; purified by ether precipitation	TFA, H_2_O, and TFA/acetonitrile	pH-responsive: open form (CBMA-OH) ↔ closed lactone (CBMA-Ring); ultralow protein adsorption from plasma	Cao et al. [28]
**CBMA-EE**	~90%	Reaction of ethyl bromoacetate with DMAEMA in acetonitrile; precipitated and washed with ether	Acetonitrile, DMF; less soluble in water before hydrolysis	Hydrolyzes in aqueous or basic conditions to zwitterionic CBMA(1)	Cheng et al. [29]
**CBMA-EE SA**	~90%	Anion exchange: bromide counter-ion replaced with salicylate using sodium salicylate	Water, ethanol/water, and ethylene glycol	Hydrolyzes to zwitterionic CBMA(1) and releases salicylate as antimicrobial; provides dual nonfouling + bactericidal function	Cheng et al. [30]

## Data Availability

No new data were created or analyzed in this study. Data sharing is not applicable to this article.

## Abbreviations

The following abbreviations are used in this manuscript:

γ-APS	3-aminopropyltriethoxysilane
ACVA	4,4′-azobis(4-cyanovaleric acid)
ADA	2,2-di(acryloyloxy-1-ethoxy)propane
AIBN	Azobisisobutyronitrile
ALCAM	Activated leukocyte cell adhesion molecule
APS	Ammonium persulfate
ATRP	Atom transfer radical polymerization
AuNRs	Gold nanorods
BIBB	2-bromoisobutyryl bromide
bpy	2,2′-bipyridine
BrTMOS	Trimethoxysilane
BSA	Bovine serum albumin
CBAAs	Polycarboxybetaine acrylamides
CBMA	Carboxybetaine methacrylate
CBMAX	Carboxybetaine dimethacrylate
CPADB	4-cyanopentanoic acid dithiobenzoate
CuBr	Copper bromide
CuBr2	Copper (II) bromide
CAC	Critical aggregation concentration
β-CD	β-cyclodextrin
CMEDTB	2-cyano-2-methylethyl dithiobenzoate
CTA	Chain transfer agent
CT	α-chymotrypsin
CTCs	Circulating tumor cells
Da	Dalton
DLC	Drug-loading content
DLE	Drug-loading efficiency
DMAEMA	2-(dimethylamino)ethyl methacrylate
DMF	Dimethylformamide
DMSO	Dimethyl sulfoxide
DNA	Deoxyribonucleic acid
DOPA	3,4-dihydroxy-L-phenylalanine
DOX	Doxorubicin
DP	Degree of polymerization
DTT	Dithiothreitol
EBIB	Ethyl 2-bromoisobutanoate
EDC	N-(3-dimethylaminopropyl)-N′-(ethylcarbodiimide hydrochloride)
EGDMA	Ethylene glycol dimethacrylate
EMAAB	4-ethoxy-4′-methacrylamide
FBS	Fetal bovine serum
FHEA	2-(perfluorohexyl)ethyl acrylate
FXII900	Factor XII inhibitor
GLBT	2-((2-(methacryloyloxy)ethyl)dimethylammonio)-acetate
GMA	Glycidyl methacrylate
GOx	Glucose oxidase
GPC	Gel permeation chromatography
HEMA	2-hydroxyethyl methacrylate
HMTETA	1,1,4,7,10,10-Hexamethyltriethylenetetramine
HUVECs	Human umbilical vein endothelial cells
IgG	Immunoglobulin G
IPN	Interpenetrating polymer network
LGA	d,l-lactide-co-glycolide
LK7	Ac-LVFFARK-NH2
MAEL	2-(methacryloyloxy) ethyl lipoate)
MBAA	N,N′-methylenebisacrylamide
MD	Molecular dynamics
MNPs	Monodispersed metal nanoparticles
MOF	Metal–organic framework
NDMCC	N,N′-dimethacryloylcystine
NHS	N-hydroxysuccinimide
NIPAM	N,N-isopropylacrylamide
PAC	Powdered activated carbon
PANI	Polyaniline
PBMA	Poly(n-butyl methacrylate)
PBS	Phosphate-buffered saline
PCBMA	Poly(carboxybetaine methacrylate)
PCL	Poly(ε-caprolactone)
PCMA	Poly(phosphorylcholine methacrylate)
PDPA	Poly(2-(diisopropylamino)ethyl methacrylate)
PEG	Polyethylene glycol
PEGDMA	Poly(ethyleneglycol) dimethacrylate
PEGMA	Poly(oligoethylene glycol methacrylate)
PEHA	Poly(ethylhexyl acrylate)
PEI	Polyethylenimine
PEMAAB	Poly(4-ethoxy-4′-methacrylamide)
PES	Polyethersulfone
PET	Photoinduced electron transfer
PFHEA	Poly[2-(perfluorohexyl)ethyl acrylate]
PHPMA	Hydroxypropyl methacrylamide
PHTE	N-propynoyl-hydrazinecarboxylic acid tert-butyl ester
PMDETA	N,N,N′,N″,N″-pentamethyldiethylenetriamine
PMMA	Poly(methyl methacrylate)
PMPC	Poly(2-(methacryloyloxy) ethyl phosphorylcholine)
PLA	Poly(l-lactide)
PLGA	Poly(lactic-co-glycolic acid)
PSBMA	Poly(sulfobetaine methacrylate)
PVA	Polyvinyl alcohol
PVP	Poly(N-vinylpyrrolidone)
RAFT	Reversible addition–fragmentation chain transfer
RGD	Arginine–Glycine–Aspartic acid–D-Phenylalanine–Lysine
RhB-HEMA	Rhodamine B-labeled 2-hydroxyethyl methacrylate
SAMs	Self-assembled monolayers
SEC	Size exclusion chromatography
SNPs	Silica nanoparticles
SP	Sodium pyruvate
SPR	Surface plasmon resonance
t-BuCBMA	2-tert-butoxy-N-(2-(methacryloyloxy)ethyl)-N,N-dimethyl-2-oxoethanaminium
TEGDMA	Triethylene glycol dimethacrylate
TEMED	N,N,N′,N′-tetramethylethylenediamine
TFA	Trifluoroacetic acid
TFE	2,2,2-trifluoroethanol
THF	Tetrahydrofuran
TMDP	4,4-trimethylene dipiperidine
